# Detecting cell-type-specific allelic expression imbalance by integrative analysis of bulk and single-cell RNA sequencing data

**DOI:** 10.1371/journal.pgen.1009080

**Published:** 2021-03-04

**Authors:** Jiaxin Fan, Xuran Wang, Rui Xiao, Mingyao Li

**Affiliations:** 1 Department of Biostatistics, Epidemiology and Informatics, University of Pennsylvania Perelman School of Medicine, Philadelphia, Pennsylvania, United States of America; 2 Department of Statistics and Data Science, Carnegie Mellon University, Pittsburgh, Pennsylvania, United States of America; New York Genome Center & Columbia University, UNITED STATES

## Abstract

Allelic expression imbalance (AEI), quantified by the relative expression of two alleles of a gene in a diploid organism, can help explain phenotypic variations among individuals. Traditional methods detect AEI using bulk RNA sequencing (RNA-seq) data, a data type that averages out cell-to-cell heterogeneity in gene expression across cell types. Since the patterns of AEI may vary across different cell types, it is desirable to study AEI in a cell-type-specific manner. Although this can be achieved by single-cell RNA sequencing (scRNA-seq), it requires full-length transcript to be sequenced in single cells of a large number of individuals, which are still cost prohibitive to generate. To overcome this limitation and utilize the vast amount of existing disease relevant bulk tissue RNA-seq data, we developed BSCET, which enables the characterization of cell-type-specific AEI in bulk RNA-seq data by integrating cell type composition information inferred from a small set of scRNA-seq samples, possibly obtained from an external dataset. By modeling covariate effect, BSCET can also detect genes whose cell-type-specific AEI are associated with clinical factors. Through extensive benchmark evaluations, we show that BSCET correctly detected genes with cell-type-specific AEI and differential AEI between healthy and diseased samples using bulk RNA-seq data. BSCET also uncovered cell-type-specific AEIs that were missed in bulk data analysis when the directions of AEI are opposite in different cell types. We further applied BSCET to two pancreatic islet bulk RNA-seq datasets, and detected genes showing cell-type-specific AEI that are related to the progression of type 2 diabetes. Since bulk RNA-seq data are easily accessible, BSCET provides a convenient tool to integrate information from scRNA-seq data to gain insight on AEI with cell type resolution. Results from such analysis will advance our understanding of cell type contributions in human diseases.

## Author summary

Detection of allelic expression imbalance (AEI), a phenomenon where the two alleles of a gene differ in their expression magnitude, is a key step towards the understanding of phenotypic variations among individuals. Existing methods detect AEI using bulk RNA sequencing (RNA-seq) data and ignore AEI variations among different cell types. Although single-cell RNA sequencing (scRNA-seq) has enabled the characterization of cell-to-cell heterogeneity in gene expression, the high costs have limited its application in AEI analysis. To overcome this limitation, we developed BSCET to characterize cell-type-specific AEI using the widely available bulk RNA-seq data by integrating cell-type composition information inferred from scRNA-seq samples. Since the degree of AEI may vary with disease phenotypes, we further extended BSCET to detect genes whose cell-type-specific AEIs are associated with clinical factors. Through extensive benchmark evaluations and analyses of two pancreatic islet bulk RNA-seq datasets, we demonstrated BSCET’s ability to refine bulk-level AEI to cell-type resolution, and to identify genes whose cell-type-specific AEIs are associated with the progression of type 2 diabetes. With the vast amount of easily accessible bulk RNA-seq data, we believe BSCET will be a valuable tool for elucidating cell type contributions in human diseases.

## Introduction

Allelic expression imbalance (AEI) refers to the phenomenon where the expression between the paternal and maternal alleles of a gene differs in their magnitude in a diploid individual [1]. In the presence of *cis*-regulatory effect, the expression increasing allele at a *cis*-regulatory polymorphism can lead to higher expression of one allele compared to the other. Such allelic imbalance in gene expression may associate with phenotypic variations among individuals and contribute to human disease. Since AEI measures the expression of two alleles expressed in the same cellular environment, evidence of AEI can rule out the influence of *trans*-regulatory variants, and thus is ideal for detecting *cis*-regulatory effects and pinpointing causal variants for disease [2].

Traditionally, AEI is studied by bulk RNA sequencing (RNA-seq) in which the allelic expression differences at heterozygous exonic single nucleotide polymorphisms (SNPs) are characterized by allele-specific read counts [3–5]. However, solid tissue is typically composed of cells that originate from diverse cell types, and bulk RNA-seq can only measure the average expression across all cells in a bulk tissue sample. Previous studies have shown that gene expression is often altered in a cell-type-specific manner, and it is possible that only certain cell types are responsible for phenotypic changes [6,7]. To gain further insight into the *cis*-regulatory effect, it is necessary to characterize AEI with cell type resolution.

Recent advances in single-cell RNA sequencing (scRNA-seq) technologies have enabled researchers to characterize individual cells and study gene expression with cell type resolution even for rare cell populations [8]. However, to study the cell-type-specific effect of AEI across individuals, full-length transcript sequencing in single cells across a large number of individuals is needed. Given the current cost of scRNA-seq, it is still cost prohibitive to generate such data. Since a large amount of bulk RNA-seq data in disease relevant tissues have already been generated in previous studies, it is desirable to characterize cell-type-specific AEI using these existing data by leveraging cell-type information provided by scRNA-seq.

Efforts have been made to deconvolve cell-type-specific effect in bulk RNA-seq data by incorporating cell type proportions as covariates. For example, Donovan *et al*. [9] and Kim-Hellmuth *et al*. [10] incorporated the interaction term between SNP genotype and cell type proportion into the traditional regression model to test for the presence of cell-type-specific expression quantitative trait loci (eQTL). However, both methods only considered the interaction effect for one cell type at a time. Failure to simultaneously account for other cell types in the model will make it difficult to interpret the findings because the identified cell-type-specific eQTL is not necessarily specific to the test cell type. The EAGLE method [11], which initially aimed to detect the association between AEI and environmental factors, can be modified to test cell-type-specific AEI by substituting the environmental factors with cell type proportions. However, similar to Donovan *et al*. [9] and Kim-Hellmuth *et al*. [10], the current EAGLE [11] model can only test for one cell type at a time. Effective methods that can simultaneously study cell-type-specific AEI across cell types are still needed.

To overcome the above-mentioned limitations, we develop a two-step regression-based procedure that can integrate **B**ulk and **S**ingle-cell RNA-seq data to detect **CE**ll-**T**ype-specific allelic expression imbalance (BSCET) simultaneously across cell types. First, we perform cell-type deconvolution analysis to infer cell type compositions in bulk RNA-seq data. Second, given estimated cell type proportions in bulk RNA-seq samples, we test cell-type-specific AEI using allele-specific bulk RNA-seq read counts. Since the degree of AEI may vary with disease phenotypes, we further extend BSCET to incorporate clinical factors such as disease status to infer covariate effects on cell-type-specific AEI. Through comprehensive benchmark evaluations and real data applications to two pancreatic islet bulk RNA-seq datasets, we show that BSCET is able to detect cell-type-specific AEI and differential cell-type-specific AEI between healthy and diseased samples.

## Results

### Overview of methods and evaluation

**Fig 1:**
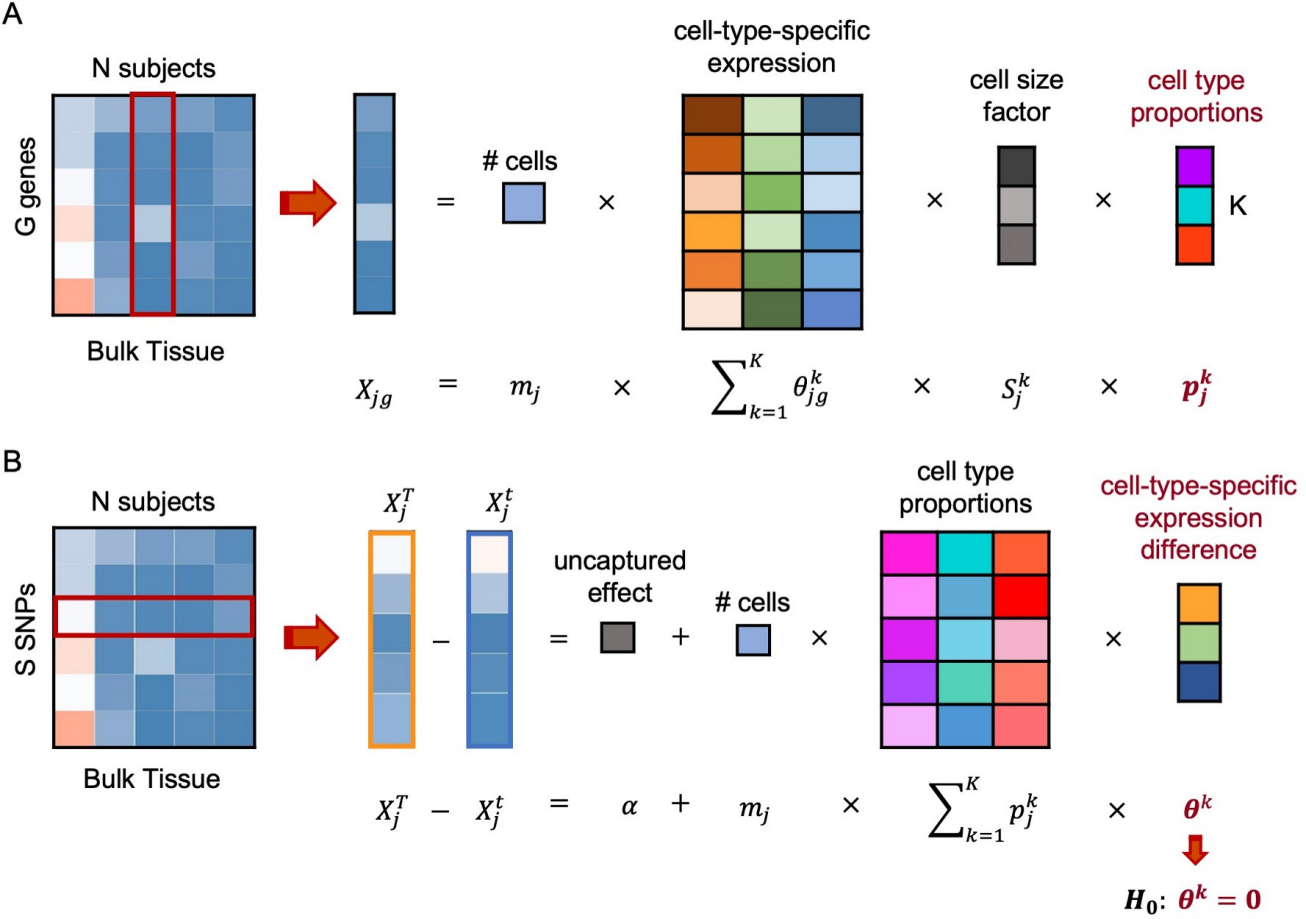
Overview of BSCET. The method contains two steps. **(A)** First, we employ the deconvolution method, such as MuSiC [14], to infer the cell type proportion, $p_{j}^{k}$, of the bulk tissue for each individual *j* of cell type *k* by incorporating the cell-type-specific expression information, $\theta_{jg}^{k}$, and cell size factor, $S_{j}^{k}$, obtained from scRNA-seq data. **(B)** Second, at each transcribed SNP (*tSNP*) site, we align the *tSNP* alleles with respect to its reference and alternative alleles, T and t, across individuals, and model the expression difference between two allele-specific read counts, $X_{j}^{T}$ and $X_{j}^{t}$, over the estimated cell type compositions, $p_{j}^{k}$, through a linear model with an intercept term *α* to capture the information not explained by the selected *K* cell types, where both *m*_*j*_ and $S_{j}^{k}$ are assumed known. The difference of population-level relative abundance between two transcribed alleles, *θ*^*k*^, can therefore be inferred for each cell type. By testing for *H*_0_: *θ*^*k*^ = 0, we can detect cell-type-specific allelic expression imbalance across samples.

The primary goal of BSCET is to detect cell-type-specific AEI across individuals using bulk RNA-seq data. It takes two datasets as input, a bulk RNA-seq dataset, which is used to detect cell-type-specific AEI, and a scRNA-seq dataset that is used as a reference to infer cell type compositions in the bulk data. After cell type compositions in all bulk RNA-seq samples are inferred, for each transcribed SNP, BSCET then aggregates allele-specific read counts from the bulk RNA-seq data across individuals to model cell-type-specific expression difference between two alleles by linear regression, and tests for the evidence of cell-type-specific AEI. For a SNP with cell-type-specific AEI, it might be of interest to further investigate if its cell-type-specific AEI is affected by any clinical factors. This can be achieved by including covariates in the regression. Specifically, BSCET models the difference in expression between the two transcribed alleles of the SNP over cell type compositions and includes interactions between cell type compositions and the covariate of interest. By testing the interaction term, BSCET assesses whether this covariate alters the cell-type-specific AEI in the corresponding cell type. An overview of BSCET is shown in **Fig 1**, and details can be found in **Methods**.

**Fig 2:**
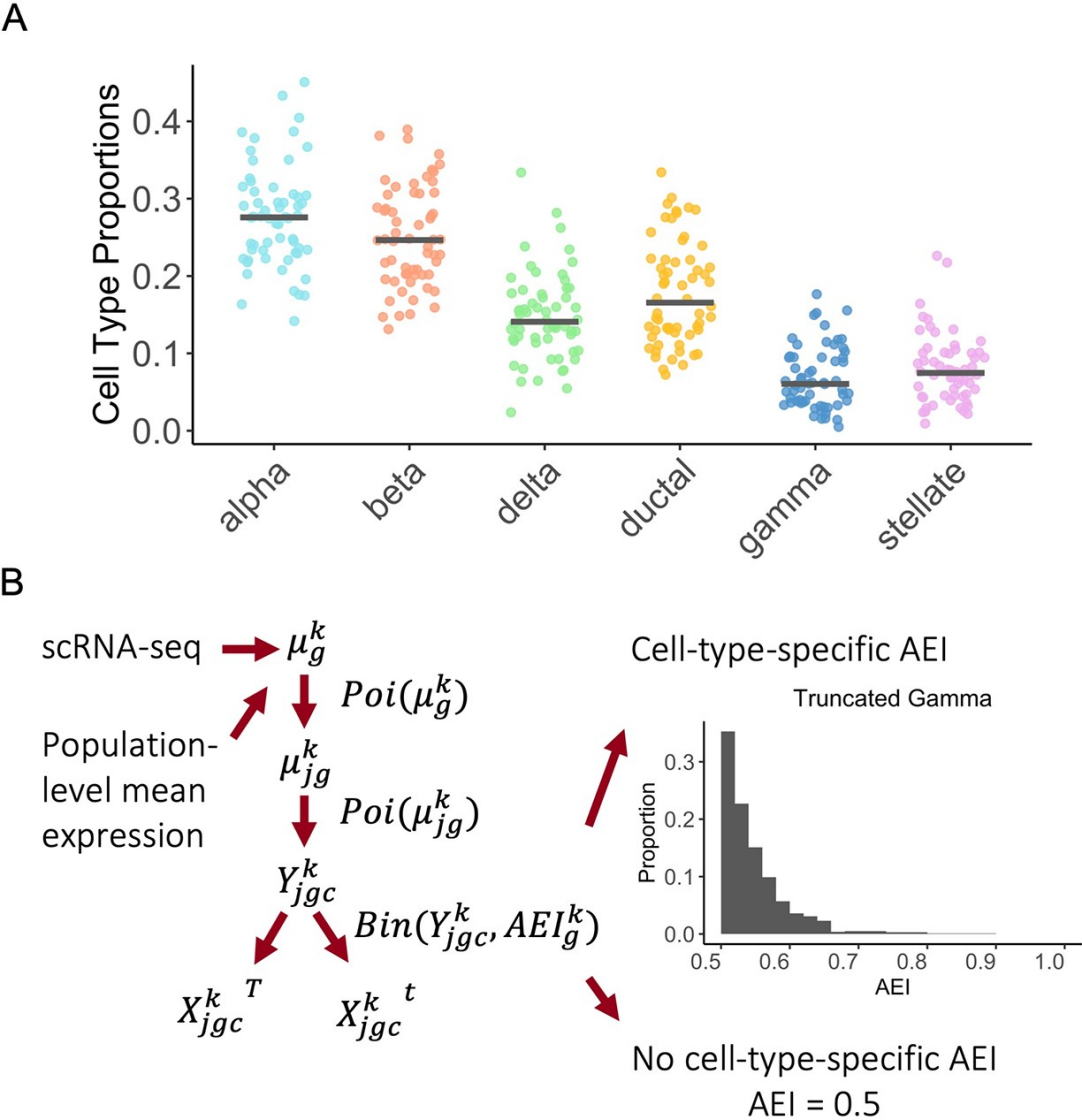
Benchmark evaluation data generation scheme. **(A)** Cell type compositions assumed for the artificial bulk RNA-seq data. For each cell type, the solid line indicates the median cell type proportions across samples, which was inferred from the single cell data [12]. **(B)** Data generation scheme for obtaining the artificial allele-specific bulk RNA-seq data. Given gene *g*, for each cell type *k*, we first inferred the mean expression level from the single cell data [12]. Based on the sample mean, we next sampled the subject-specific mean expression through Poisson distribution, and used another layer of Poisson sampling to obtain the total read count of each cell *c*. The total expression was then split into two allele-specific read counts through a binomial distribution with the probability equals to the cell-type-specific AEI. For each gene *g*, we generated data only for a single SNP, and the level of AEI equaled to 0.5 for cell types with no allelic imbalance and was generated from a truncated gamma distribution for cell types with AEI. The artificial bulk RNA-seq data was obtained by summing up allelic read counts across all cells.

We performed benchmark simulations to evaluate the performance of BSCET. The benchmark bulk RNA-seq data were generated based on a human pancreatic islet scRNA-seq dataset [12]. For the simulated benchmark data, the cell type proportions and AEI levels for each cell type are known, which allow us to evaluate the performance of BSCET. The benchmark simulation procedure was shown in **Fig 2** and details were described in **Methods**. As a comparison, we also analyzed the benchmark data using a traditional bulk AEI detection method that ignores cell-type-specific effect. In this analysis, the allelic read counts were modeled using a generalized linear model (GLM) for binomial data with logit link function. The relative expression of the reference allele over total read counts was modeled across individuals using an intercept only model, and evidence of AEI was assessed by testing whether the intercept is significantly different from zero.

### Detection of cell-type-specific AEI when one cell type has AEI

The simulated benchmark bulk RNA-seq data include 60 heterozygous individuals and the data were generated with known cell type proportions and cell-type-specific AEI levels. We considered two scenarios: first, we assumed only the major cell type, had AEI; and second, we assumed both the major and a non-major cell type, had AEI. For each gene, the major cell type was selected as the cell type with the highest mean expression among all cell types, and a non-major cell type was selected as the one with median mean expression in the original scRNA-seq data in which the benchmark bulk RNA-seq data were sampled from. Under each scenario, the model performance was evaluated using both the true and “estimated” cell type proportions, where the “estimated” proportions were obtained by adding random noise to the true proportions to reflect estimation uncertainty. More specifically, for individual *j* of cell type *k*, the “estimated” proportion, ${\hat{p}}_{j}^{k}$, was set as ${\hat{p}}_{j}^{k}=p_{j}^{k}(1+\varepsilon_{jk})$, where $p_{j}^{k}$ is the true proportion and the random noise, *ε*_*jk*_, was sampled from *N*(0,0.2^2^).

**Fig 3:**
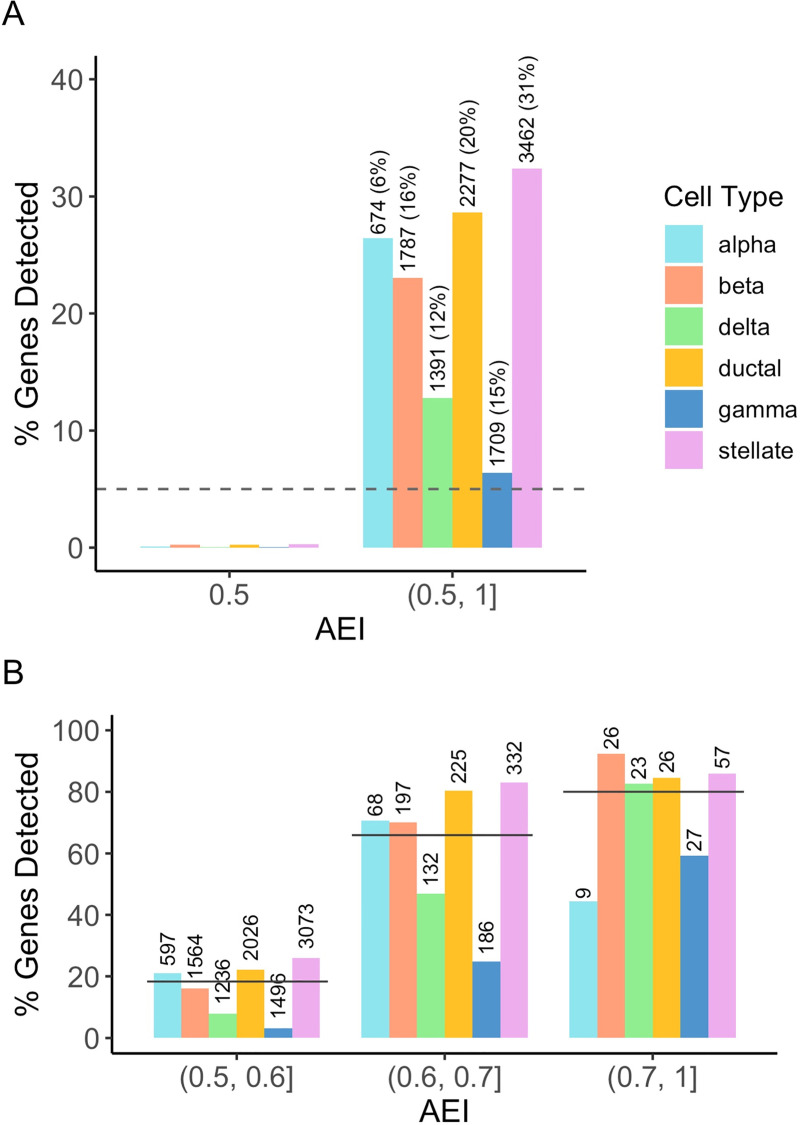
Benchmark evaluation for cell-type-specific AEI detection assuming one cell type with AEI. We evaluated the performance of BSCET assuming the major cell type for each SNP, i.e., cell type with the highest mean expression in scRNA-seq data [12], had cell-type-specific AEI using true cell type proportions. **(A)** Type I error rate and power evaluated at the cell type resolution under significance level *α* = 0.05 (dashed line). The number and percentage above each bar indicates number of SNPs, out of 11,300 tested SNPs, with cell-type-specific AEI of each cell type. **(B)** Detection power separated by the cell type and true level of AEI. The number above each bar indicates the total number of SNPs with cell-type-specific AEI within the category. The solid line indicates the overall power, i.e., across all cell types, at each level of AEI.

We analyzed 11,300 SNPs in total and evaluated the type I error on cell types that did not have cell-type-specific AEI. Under the first scenario where only the major cell type had AEI, among all 11,300 tested SNPs, 674 SNPs (6%) were selected as having cell-type-specific AEI for alpha cells, 1787 SNPs (16%) for beta cells, 1391 SNPs (12%) for delta cells, 2277 SNPs (20%) for ductal cells, 1709 SNPs (15%) for gamma cells, and 3462 SNPs (31%) for stellate cells. When using true cell type proportions as input for BSCET, the false positives were under control at the 0.05 significance level (**Fig 3A**). We correctly identified 2,650 SNPs (23%) showing cell-type-specific AEI after false discovery rate (FDR) adjustment for multiple testing. The relatively low overall detection power was mainly due to the fact that we assumed a relatively small cell-type-specific AEI for the majority of the tested SNPs. The overall detection power was higher for alpha, beta and ductal cells than delta and gamma cells, which is not surprising since alpha, beta and ductal cells are the common cell types in islets [13,14]. Interestingly, despite the low proportion in bulk tissue, stellate cells had the highest detection power among all cell types. We found that, among SNPs with AEI, the cell-type-specific mean expression levels for stellate cells were higher than the other cell types in the benchmark data. Moreover, among SNPs with AEI detected, more SNPs showed much higher cell-type-specific expression in stellate cells as compared to other cell types (**S1A Fig**). We further quantified the expression for each cell type by molecular proportion, calculated as the mean cell-type-specific expression multiplied by cell type proportion. We found that, although with some outlier SNPs, the stellate cells did not have higher molecular proportion than other cell types, suggesting that the detection power of BSCET is more likely driven by cell-type-specific mean expression, rather than by the molecular proportions (**S1B Fig**). As a comparison, the traditional GLM method for bulk data analysis detected 2,819 SNPs (25%) with AEI, among which 1,661 overlapped with the SNPs detected by BSCET. We found that SNPs detected only by the bulk GLM method had a higher expression level on average (mean reads of 481.7 with interquartile range (IQR) 166.9–583.3) compared to those detected only by BSCET (mean reads of 144.2 with IQR 55.4–183.3) (**S2 Fig**). As the bulk detection method is sensitive to high read counts, whereas BSCET deconvolves the read counts into each cell type, it is not surprising that the bulk method is more powerful than BSCET when sequencing depth is high. However, the goal of BSCET is not to detect more genes than the bulk GLM method, but rather to elucidate which cell type is driving the evidence of AEI. Nevertheless, for SNPs with not that high coverage at the bulk level, BSCET correctly detected some of them with cell-type-specific AEI while the bulk GLM method failed to detect. For most of these SNPs, the major cell type does not have high cell type proportions, thus the bulk GLM method has limited detection power as the AEI signals were diluted due to other more common cell types that do not have AEI.

We further evaluated the performance of BSCET at different AEI levels (**Fig 3B**). As expected, BSCET detected more genes as the true AEI level increased, and the power reached 80% when the AEI was as large as 0.7. Similarly, the power increased with AEI level for most cell types, except for alpha cells, which was likely due to the relatively small number of SNPs in the [0.7, 1) AEI category. Further, at different AEI levels, alpha, beta, ductal and stellate cells had higher detection power in general than delta and gamma cells, and the difference became smaller as the underlying true AEI increased. BSCET performed the best in stellate cells when the AEI effect was low to moderate, indicating that it is able to detect small AEI effect even for rare cell types, as long as the cell-type-specific expression level is relatively high.

Next, we evaluated the performance of BSCET using “estimated” cell type proportions and the correlation between the true and “estimated” cell type proportions can be found in **S3A Fig**. Again, the type I error was well controlled. Among the 11,300 tested SNPs, 2,274 (20%) were detected to have cell-type-specific AEI after FDR adjustment. As expected, the power was slightly lower than that when the true cell type compositions were used. However, the SNP-level p-values obtained using the “estimated” proportions were highly correlated with those obtained using the true proportions (Pearson’s correlation coefficient R = 0.94, **S3B Fig**). Further, we observed a similar increasing pattern in power over the true AEI levels as well as similar power differences between cell types, indicating that BSCET is robust to estimation uncertainty in cell type proportions (**S3C Fig**).

### Detection of cell-type-specific AEI when two cell types have AEI

**Fig 4:**
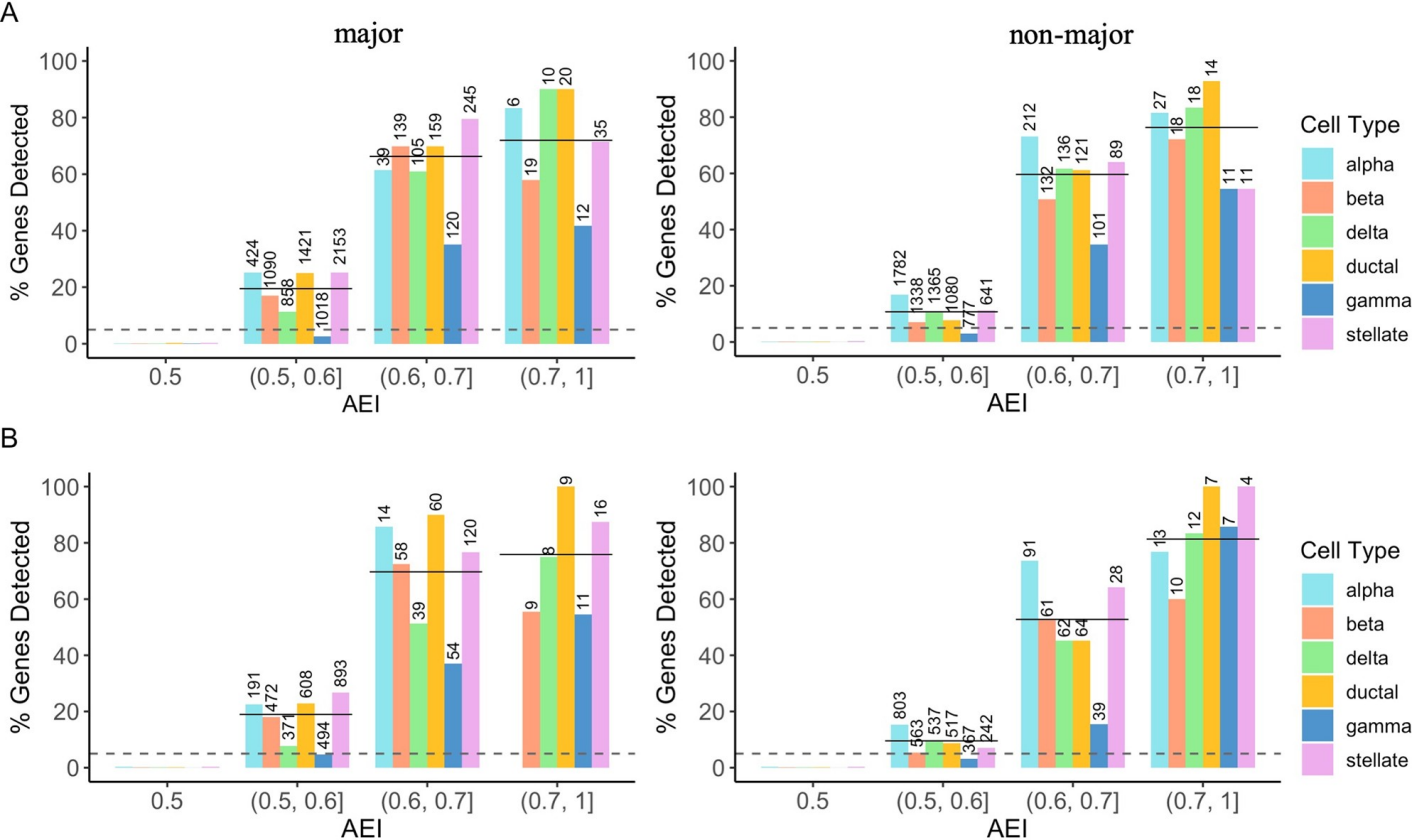
Benchmark evaluation for cell-type-specific AEI detection assuming two cell types with AEI. We evaluated the performance of BSCET assuming both the major and a non-major cell types had AEI using true cell type proportions. For each gene, the major cell type was selected as the one with the highest mean expression, and a non-major cell type was selected as the one with median mean expression in scRNA-seq data [12]. The AEI level for two cell types were assumed to be in the same direction, i.e., their AEI levels are > 0.5 for both cell types, for 70% of the SNPs, and in opposite directions, i.e., their AEI levels sum to 1, for the remaining 30% of the SNPs. **(A)** Type I error rate and power evaluated separately by cell type and true level of AEI for the 70% SNPs with the same direction of AEI under significance level *α* = 0.05 (dashed line) for the major **(left)** and non-major cell types **(right)**. **(B)** Type I error rate and power evaluated separately by cell type and true level of AEI for the 30% SNPs with opposite directions of AEI under significance level *α* = 0.05 (dashed line) for the major **(left)** and non-major cell types **(right)**. For each plot, the number above each bar indicates the total number of SNPs with cell-type-specific AEI within the category. The solid line indicates the overall power across all cell types.

We also considered the scenario in which both the major cell type and a non-major cell type had AEI. Rather than letting all SNPs had AEI for both cell types in the same direction, we assumed the effect was in opposite directions for 30% of the 11,300 tested SNPs. For these 30% of the SNPs, the AEI effects in the two cell types would cancel each other out when averaging across all cells, thus the bulk detection method might fail to detect evidence of AEI. Assuming the true cell type compositions were known, among the 7,873 (70%) tested SNPs with AEI in the same direction, BSCET correctly uncovered 1,920 (24%) for the major cell type and 1,268 (16%) for the non-major cell type, with type I error well controlled at the 0.05 significance level. For the major cell type, the results were similar to those obtained under the first scenario, indicating that the performance of BSCET was not influenced much when more than one cell type had AEI. For the non-major cell type, due to its lower expression level than the major cell type, the detection power, as expected, was lower than that for the major cell type. Nevertheless, we still had around 80% power when the underlying AEI was large, e.g., over 0.7, and the power difference between the major and non-major cell types decreased as the AEI level increased. Among the 3,427 (30%) tested SNPs with opposite AEI directions, we correctly recovered the direction of cell-type-specific AEI for 2,330 SNPs (68%), and identified 837 (24%) with AEI for the major cell type and 502 (15%) for the non-major cell type after FDR adjustment. For both the major and non-major cell types, the patterns of type I error and power are consistent with those observed in **Fig 4A**, suggesting that BSCET was reliable for detecting cell-type-specific effect even when the two alleles of the transcribed SNP were regulated differently across cell types (**Fig 4B**).

In comparison, among the 7,873 SNPs with AEI in the same direction, the bulk GLM method correctly identified 4,840 (61%) SNPs with AEI, of which 2,609 overlapped with findings by BSCET. Since bulk-level AEI reflects the collective effect of AEI across all cell types, the bulk GLM method is expected to be more powerful when the goal is to detect the overall evidence of AEI. But still, BSCET was able to detect an additional 578 SNPs with cell-type-specific AEI for the major cell type and 422 SNPs for the non-major cell type that were missed by the bulk method. For the 3,427 SNPs with opposite AEI effects, the bulk GLM method detected 644 (18%) SNPs with AEI, and 444 of them overlapped with those detected by BSCET. This time, the bulk detection method was less powerful than BSCET, which was not surprising because the cell-type-specific AEI was neutralized when expression was averaged across cell types. For SNPs with bulk AEI level between 0.45 and 0.55, BSCET correctly uncovered many of them showing cell-type-specific AEI in both the major and non-major cell types, while the GLM model failed to detect any, indicating that BSCET was able to reveal cell-type-specific effect when the AEI was masked at the bulk level (**S4 Fig**). When repeating the same analysis but using the “estimated” cell type proportions as input for BSCET, for the 70% SNPs with the same AEI direction, we identified 1,766 (22%) SNPs for the major cell type and 1,119 (14%) for the non-major cell type after FDR adjustment, respectively. For the 30% SNPs with opposite AEI effects, BSCET correctly detected 707 (21%) for the major cell type and 375 (11%) for the non-major cell type. As expected, estimation uncertainty in cell type proportions slightly decreased BSCET’s detection power. But overall, the results were consistent with those using the true cell type proportions as input with the correlation of p-values over 0.9 (**S5 Fig**).

### Detection of cell-type-specific differential AEI

**Fig 5:**
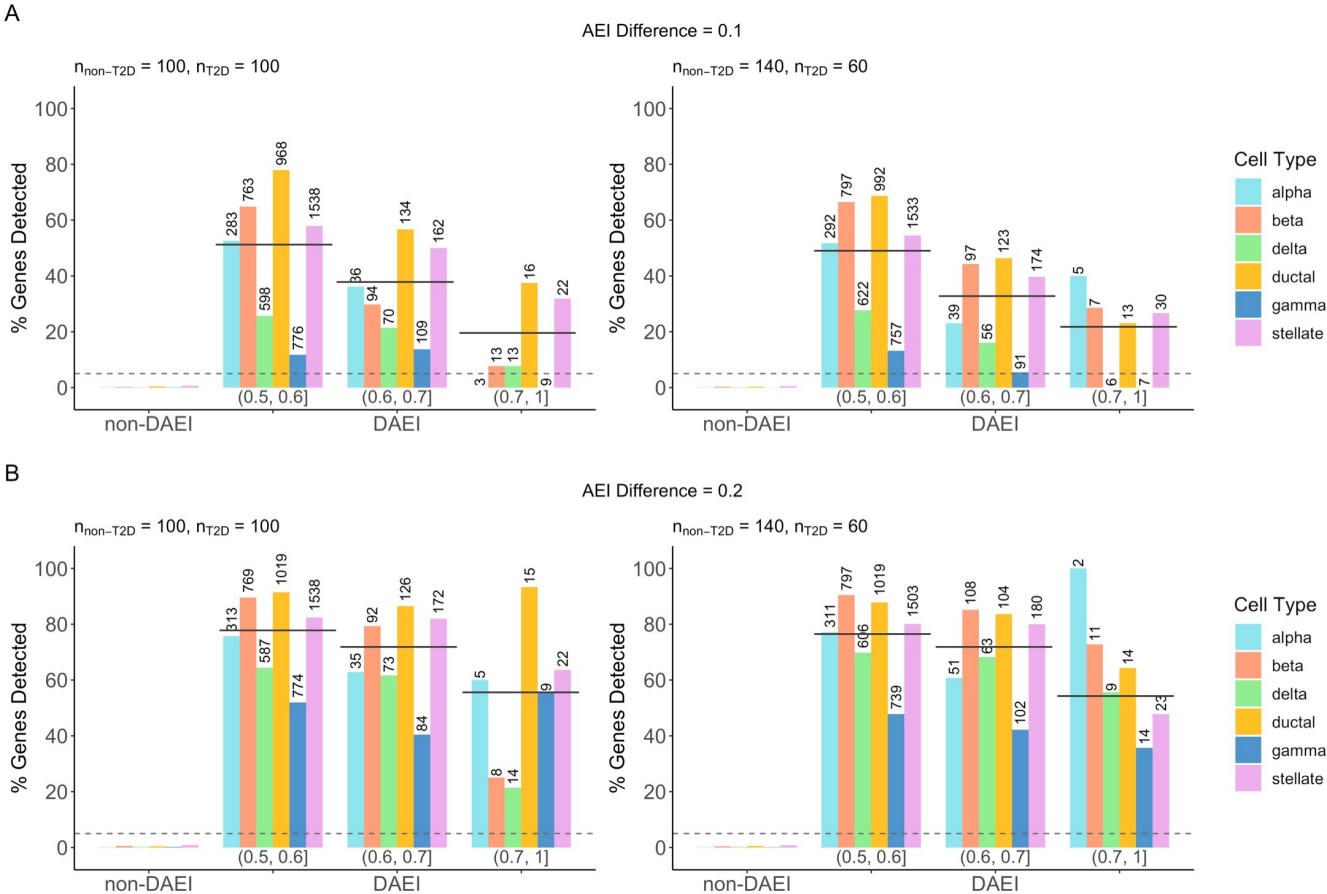
Benchmark evaluation for cell-type-specific differential AEI (DAEI) detection. Using true cell type proportions, we evaluated the performance of BSCET as a function of sample size for healthy (i.e., non-T2D) and diseased (i.e., T2D) samples, and true cell-type-specific AEI difference between two-condition samples (0.1 **(A)** and 0.2 **(B)**). Type I error rate (non-DAEI) and power (DAEI) were evaluated at significance level *α* = 0.05 (dashed line), further separated by the cell type and level of AEI in the healthy samples. The number above each bar indicates the total number of SNPs with cell-type-specific DAEI within the category. The solid line indicates the overall power across all cell types.

We further evaluated the performance of BSCET for detecting covariate effect on cell-type-specific AEI. We considered a case-control setting and assessed the effect of disease status on AEI, i.e., differential AEI (DAEI) between healthy and diseased samples. First, we considered equal number of cases and controls, i.e., non-diabetes and diabetes given the pancreatic islets data setting (100 vs. 100). **Fig 5** shows that BSCET did not generate excessive false positive results for SNPs without cell-type-specific DAEI at the 0.05 significance level. When the difference of cell-type-specific AEI between cases and controls was small, e.g., 0.1, using true cell type proportions as input, among all 5,607 SNPs with cell-type-specific DAEI, BSCET identified 2,776 (50%) of them after multiple testing adjustment, whereas the bulk detection method uncovered 3,199 (57%) SNPs, with 2,136 overlapped with BSCET. When the differential AEI effect increased to 0.2, among the 5,655 SNPs with cell-type-specific DAEI, BSCET identified 4,370 (77%), while the bulk method identified 4,647 (82%), of which 3,981 overlapped with BSCET. Similar to one condition analysis, under both scenarios, SNPs uniquely detected by BSCET on average had lower sequencing depth as compared to SNPs uniquely detected by the bulk method. For example, when the cell-type-specific DAEI was 0.1, the mean read count for bulk-uniquely detected SNPs was 385.0 (IQR 133.4–479.8) and 98.4 (IQR 44.5–121.8) for BSCET-uniquely detected SNPs. In addition, the power decreased as the AEI level in controls increased. This was expected because given the same AEI difference between the two groups, a higher AEI level in controls led to a lower relative change of AEI between conditions.

In real studies, we often have less cases than controls. Thus, we also considered a scenario assuming 60 cases versus 140 controls. Again, the type I error was under control. Compared to results with balanced sampling, this scenario had lower detection power, but BSCET still correctly detected 2,676 (47%) out of 5,641 SNPs with cell-type-specific DAEI when the true DAEI was 0.1, and 4,317 (76%) out of 5,656 when the true DAEI increased to 0.2 (**Fig 5**). Additionally, when using “estimated” cell type proportions as input, across all scenarios, the results were highly correlated with those obtained using true cell type compositions (**S6 Fig**).

### Application to Fadista *et al*. human pancreatic islet bulk RNA-seq data

**Fig 6:**
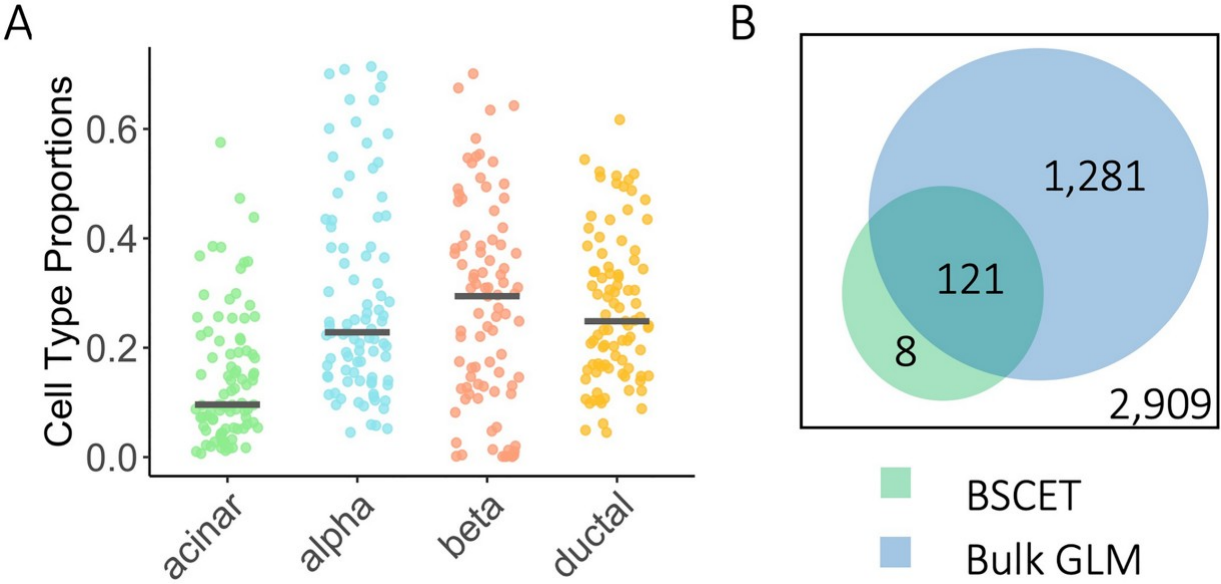
Deconvolution and cell-type-specific AEI detection of the Fadista pancreatic islets RNA-seq data. **(A)** Cell type proportion estimates of the Fadista bulk RNA-seq data [15] using MuSiC [14]. The Segerstolpe scRNA-seq data [16] were used as the reference. **(B)** Venn diagram showed the total number of genes analyzed, and number of genes detected with cell-type-specific AEI after FDR multiple testing adjustment using BSCET (green) and traditional bulk AEI detection method based on GLM model (blue).

We applied BSCET to detect cell-type-specific AEI using human pancreatic islet bulk RNA-seq data of 89 donors generated in an expression quantitative trait loci (eQTL) study by Fadista *et al*. [15]. We focused our analysis on four well-recognized cell types: acinar, alpha, beta, and ductal. We first deconvolved the bulk RNA-seq data by MuSiC [14] using the scRNA-seq data from six healthy donors generated by Segerstolpe *et al*. [16] as reference. The estimated cell type proportions were shown in **Fig 6A**. Allele-specific read counts for transcribed SNPs were obtained using WASP [17], which removes the possible mapping bias in read alignment. For each transcribed SNP, only individuals heterozygous for the locus were considered, and an individual was kept for further analysis if its minor allele count ≥ 5, total read count for both alleles ≥ 20 and minor allele count was at least 5% of the total read count. A SNP was included in the analysis if there were at least 20 individuals left after the filtering.

In total, we analyzed 5,972 SNPs across 2,909 genes using both BSCET and the bulk GLM method for AEI detection. After FDR adjustment, 283 SNPs across 129 (5%) genes were detected as having AEI by BSCET for at least one cell type (**S1 Table**). Of the 129 genes, 121 (94%) were identified while 8 (6%) were missed by the bulk method. The bulk method detected an additional 1,281 genes with AEI (**Fig 6B**). Several reasons may help explain the lack of power of BSCET in this real data analysis and the power difference between BSCET and bulk GLM model. First, Fadista data did not have a high sequencing depth, with mean total read count of 108.8 (IQR 35.1–81.7) for all tested SNPs. As BSCET deconvolves total expression to each cell type, it was not surprising that BSCET needs higher coverage to achieve a similar detection power as the bulk analysis. Second, the average number of heterozygous individuals per SNP is only 34 (IQR 25–40) for this dataset, a relatively small sample size considering the number of cell types included in the analysis, which, in turn, also explains the outperformance of bulk analysis as it is an intercept only model.

Next, we compared genes detected by BSCET with the 616 eGenes identified to have *cis*-eQTL SNPs by Fadista *et al*. [15]. Among the 616 eGenes, 56 were analyzed by BSCET and 6 (11%) had cell-type-specific AEI. To evaluate if 11% overlapping was higher than expected by chance, we performed resampling-based enrichment analysis. Specifically, we randomly sampled 129 genes from the remaining 2,780 genes that did not show evidence of cell-type-specific AEI by BSCET and recorded the percentage of genes overlapping with the 616 eGenes in Fadista *et al*. [15]. By repeating this sampling procedure 10,000 times, we calculated the enrichment p-value as $\frac{\#(p_{resampled}\geq 11\%)}{10,000}$, which equaled to 0.029, suggesting that results from BSCET significantly overlapped with the *cis*-eQTL findings. For the rest of the 123 cell-type-specific AEI genes that were not identified as eGenes, 68 (55%) were found to have AEI only in one cell type. As the cell-type-specific effect may be diluted after averaging across cells in bulk RNA-seq, this could explain the low overlapping proportion between BSCET and eQTL analysis. In addition, among the remaining 55 (45%) genes with cell-type-specific effect detected in more than one cell type, 15 (27%) were found to have AEI in opposite directions. It is likely that cell-type-specific AEI effects are canceled out at the bulk level and therefore missed by the eQTL analysis.

**Fig 7:**
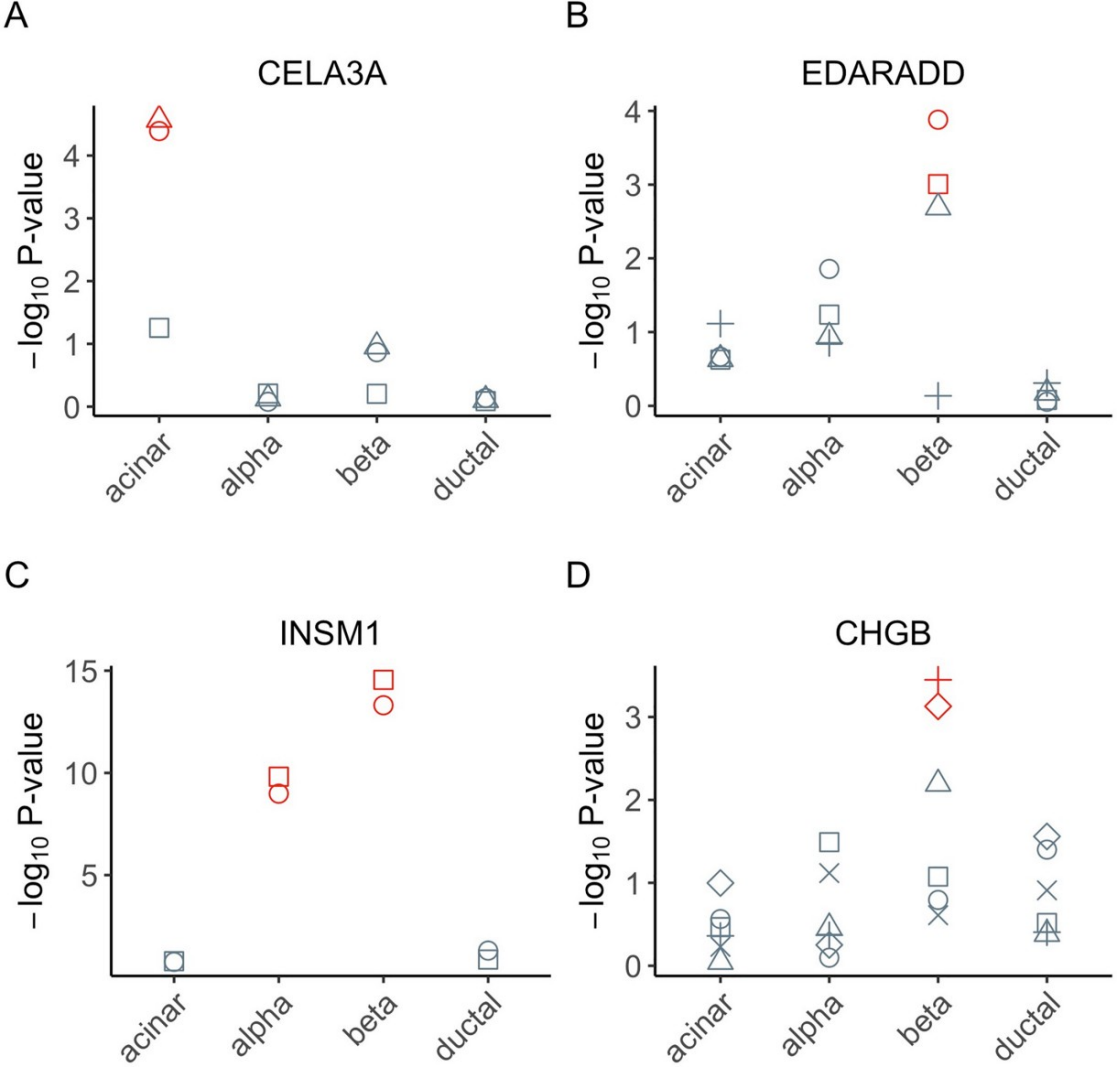
Selected genes with cell-type-specific AEI of the Fadista pancreatic islets RNA-seq data. We selected 4 genes, *CELA3A* **(A)**, *EDARADD* **(B)**, *INSM1* **(C)** and *CHGB* **(D)**, to show their SNP-level p-values of cell-type-specific AEI using the Fadista bulk RNA-seq samples [15]. The input proportion estimates were obtained using MuSiC [14] based on Segerstolpe single-cell reference [16]. Within each cell type, different shapes represent different SNPs of the gene, with red color indicating significant AEI after FDR multiple testing adjustment.

For genes identified with cell-type-specific AEI by BSCET, some of them are cell-type-specific marker genes by PanglaoDB [18]. For example, *CELA3A*, with two transcribed SNPs detected having cell-type-specific AEI by BSCET, is a marker gene for acinar cells (**Fig 7A**), and BSCET only detected AEI in acinar cells but not in the other cell types, confirming the specificity of BSCET. Furthermore, we uncovered two other marker genes for acinar cells with cell-type-specific AEI, *CPA2* and *PRSS3* (**S7 Fig**). In addition to acinar cells, we also detected a marker gene for beta cells with cell-type-specific AEI, *EDARADD*, which had consistent signals across the majority of transcribed SNPs in this gene (**Fig 7B**).

For genes identified by BSCET but are not documented as marker genes by PanglaoDB [18], we found many of them are biologically relevant. For example, *INSM1* had cell-type-specific AEI for both alpha and beta cells with signals well agreed between the two SNPs analyzed for this gene (**Fig 7C**). Several studies have reported *INSM1* as a key factor for the development and differentiation in pancreatic beta cells [19,20]. Another example is *CHGB*, which had significant cell-type-specific AEI for beta cells by BSCET (**Fig 7D**). A recent study has shown that *CHGB* is essential for regulating secretory granule trafficking in beta cells [21]. Several other genes identified by BSCET are also known to have relevant functions. For example, *PPM1K* elevates branched-chain amino acids concentrations and is related to high risk of T2D [22,23]. *SYT11* has been reported to be positively related to glucose stimulated insulin secretion in human pancreatic islets [24]. *ERO1B* is known to encode a pancreas-specific disulfide oxidase that promotes protein folding in pancreatic beta cells [25]. *AHR* is expressed in aortic endothelial cells and is activated in response to high glucose stimulation [26] (**S7 Fig**).

### Evaluation on the impact of scRNA-seq reference

To examine whether BSCET is robust to the input cell type compositions, we reanalyzed the Fadista *et al*. bulk RNA-seq data using a different scRNA-seq dataset as reference. The new scRNA-seq dataset was generated from three healthy donors by Baron *et al*. [12]. Cell type deconvolution using this scRNA-seq reference resulted in a much smaller proportion for beta cells compared to results obtained using the Segerstolpe scRNA-seq data [16] as reference (**S8A Fig**). Using the new cell type proportions as input for BSCET, we identified 148 (5%) out of 2,909 analyzed genes to have cell-type-specific AEI, among which 102 were also found in previous analysis when using the Segerstople scRNA-seq data [16] as reference (**S2 Table**). Overall, the SNP level p-values obtained from these two different scRNA-seq reference datasets were well correlated (R = 0.72, **S8B Fig**). Stratified by cell type, the correlation was the highest for alpha cells (R = 0.87), followed by acinar (R = 0.81) and ductal (R = 0.77) cells. The correlation for beta cells was moderate (R = 0.59), which can be explained by the large difference in the estimated beta cell proportions when using the two single-cell datasets as reference (mean proportion: 3% vs. 28%, **S8C Fig**). Overall, BSCET yielded relatively consistent results, suggesting that it is robust to the choice of single-cell reference for deconvolution.

### Replication using Bunt *et al*. human pancreatic islet bulk RNA-seq data

**Fig 8:**
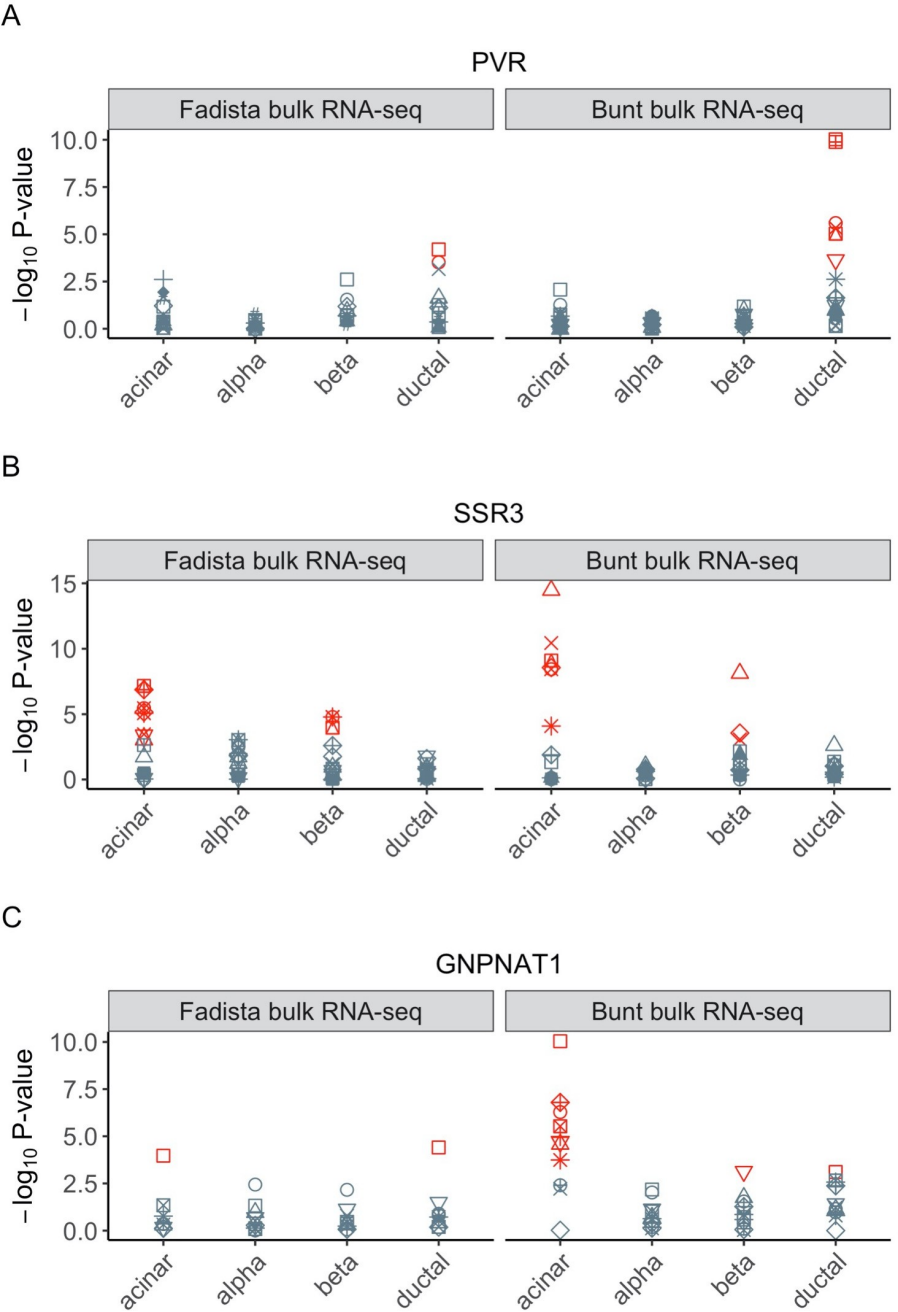
Selected genes with cell-type-specific AEI of both pancreatic islet RNA-seq data. We selected 3 genes, *PVR* **(A)**, *SSR3* **(B)** and *GNPNAT1* **(C)**, to show their SNP-level p-values of cell-type-specific AEI using two different bulk samples, Fadista *et al*. [15] and Bunt *et al*. [27]. For both samples, the input proportion estimates were obtained using MuSiC [14] based on Segerstolpe single-cell reference [16]. Within each cell type, different shapes represent different SNPs of the gene, with red color indicating significant AEI after FDR multiple testing adjustment.

We further applied BSCET to another islets RNA-seq data of an eQTL study generated from 118 individuals by Bunt *et al*. [27] using the Segerstolpe scRNA-seq data [16] as reference. The estimated cell type compositions are overall similar to the Fadista data [15], except for a smaller proportion for alpha cells (**S9 Fig**). In total, we analyzed 8,984 SNPs across 4,197 genes, with the average sample size of 37 per SNP (IQR 25–44) and mean total read count of 90 (IQR 40–88). After multiple testing adjustment, BSCET detected 678 SNPs across 405 (10%) genes showing cell-type-specific AEI (**S3 Table**). Next, we compared these results with those obtained from the Fadista data [15]. Among the 48 genes detected with AEI for acinar cells in Fadista, 41 were analyzed in Bunt, among which 23 (56%) also had AEI in acinar cells. Similarly, for the 81 genes with AEI for beta cells in Fadista, 33 (41%) were replicated in Bunt. For ductal cells, among the 27 genes with AEI in Fadista, 15 (56%) were replicated in Bunt. However, for alpha cells, among the 39 genes detected in Fadista, only 6 genes (15%) were replicated in Bunt. This is likely due to the large discrepancy in estimated cell type proportions for alpha cells between the two datasets (mean proportion: 5% vs. 29%). Despite some difference, the AEI results obtained using the Bunt data validated findings obtained in Fadista. For example, *PVR*, also known as *CD155*, involves in the regulation of T-cell activation and is associated with autoimmune diseases such as type 1 diabetes (T1D) [28,29], had two SNPs showing AEI for ductal cells in Fadista. This finding was confirmed in Bunt in multiple SNPs (**Fig 8A**). Similarly, we detected acinar and beta cell-specific AEI for several SNPs in gene *SSR3* in both datasets (**Fig 8B**). Gene *SSR3* is involved in protein translocation across the endoplasmic reticulum (ER) membrane, whose expression has showed to be related to T2D [30,31]. Moreover, gene *GNPNAT1* was detected only for one SNP with acinar cell-specific AEI in Fadista, but was confirmed with much stronger effect in Bunt (**Fig 8C**). Previous studies have shown that *GNPNAT1 is* associated with insulin secretion and diabetes [32,33].

### Association of cell-type-specific AEI with HbA1c level

**Fig 9:**
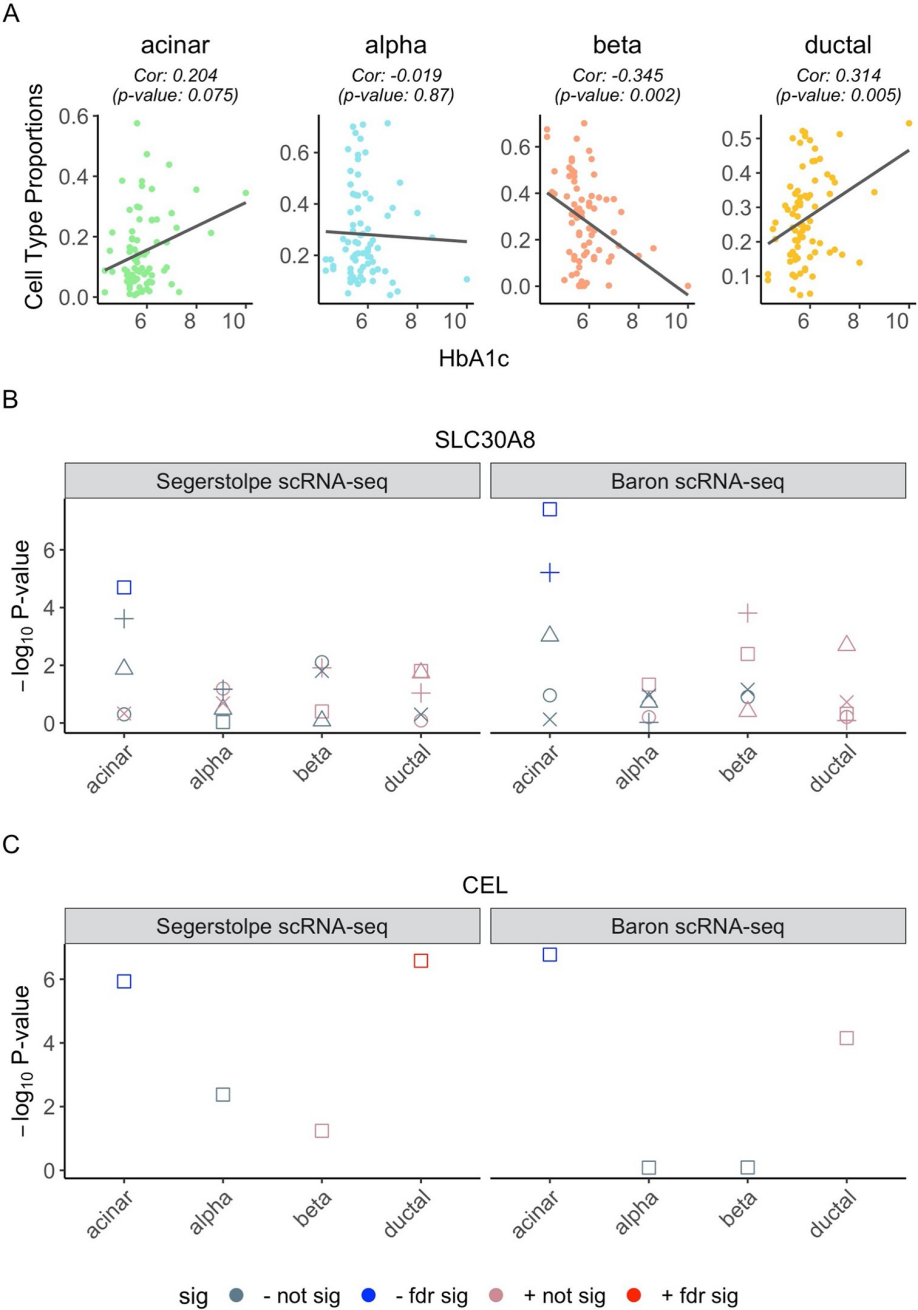
Cell type compositions and selected genes with cell-type-specific AEI associated with the progression of T2D of the Fadista pancreatic islets RNA-seq data. **(A)** Scatter plot of HbA1c vs. estimated proportions of the Fadista bulk RNA-seq data [15] for each cell type with a fitted regression line, where the proportions were estimated through MuSiC [14] based on the Segerstolpe single-cell reference [16]. Within each cell type, we calculated the Spearman’s correlation and the corresponding p-value between HbA1c and estimated cell type proportions and showed as the subtitle of the plot. **(B**,**C)** We selected 2 genes, *SLC30A8* **(B)** and *CEL* **(C)**, to show their SNP-level p-values of the association between HbA1c and cell-type-specific AEI, where we used cell type proportion estimates obtained from two different scRNA-seq reference datasets: Segerstolpe *et al*. [16] and Baron *et al*. [12]. Within each cell type, different shapes represent different SNPs. Red color indicates a positive correlation (+) between HbA1c and cell-type-specific AEI, with color brightness representing significance level. Similarly, blue color indicates a negative correlation (-) between HbA1c and cell-type-specific AEI, with color brightness representing significance level.

Type 2 diabetes (T2D) is characterized by the progressive dysfunction of pancreas islets. Next, we applied BSCET to explore the association between hemoglobin A1c (HbA1c), a well-known biomarker for T2D diagnosis, and cell-type-specific AEI. People with higher HbA1c are more likely to develop T2D [34]. Using the Fadista bulk RNA-seq data, we focused our analysis on the 77 samples with HbA1c level available. **Fig 9A** showed relationship between HbA1c level and estimated cell type proportions with Segerstolpe scRNA-seq data [16] as reference. As expected, we observed a negative association between beta cell proportion and the HbA1c level. After applying the same filtering criteria as described previously, we analyzed a total of 5,021 SNPs across 2,570 genes, and identified 8 (0.3%) genes, *HYOU1*, *PLA2G1B*, *CCDC32*, *CCL2*, *CDC42EP3*, *LARS*, *SLC30A8* and *CEL*, whose cell-type-specific AEI was associated with HbA1c level (**S4 Table**). By contrast, using the bulk AEI detection method, we detected 125 (5%) genes with significant association between AEI and HbA1C level, and among which, *LARS*, *SLC30A8* and *CEL* overlapped with those detected by BSCET.

BSCET revealed a negative association of HbA1c level with the acinar cell-specific AEI for *SLC30A8* (p-value = 0.00002) (**Fig 9B**). This is consistent with previous studies reporting that *SLC30A8* encodes an islet zinc transporter (ZnT8) and a reduced zinc transport can increase the risk of T2D [35,36]. Moreover, a human study suggested that *SLC30A8* haploinsufficiency helps protect against T2D [37]. To further validate our findings, we repeated the analysis but using the cell type composition estimated with the Baron scRNA-seq data [12] as reference (**S10A Fig**). After multiple testing adjustment, we identified 4 genes, *CCDC32*, *LARS*, *SLC30A8* and *CEL*, whose cell-type-specific AEI was associated with HbA1c level, and all were also detected when using the Segerstolpe data as single-cell reference (**S5 Table**). For *SLA30A8*, we observed a consistent negative association between acinar cell-specific AEI and HbA1c level. Similar negative association of acinar cell-specific AEI was detected for another gene, *CEL* (**Fig 9C**), a marker gene for acinar cells in PanglaoDB [18]. Previous studies have reported that *CEL* mutations can lead to childhood-onset pancreatic exocrine dysfunction and diabetes mellitus from adulthood [38,39]. For the other two genes, *CCDC32* and *LARS*, further investigations are needed as little is known about their functions related to T2D (**S10 Fig**).

## Discussion

Detection of AEI is an important step towards the understanding of phenotypic variations associated with gene expression differences across individuals. Traditionally, AEI is detected at the tissue level using bulk RNA-seq data. However, solid tissue includes cells from different cell types and gene expression variations are often cell-type-specific. Methods that detect AEI at the bulk tissue level only capture the averaged effect of gene expression across cells, making it difficult to discern what cell types are driving the AEI signal. Although it is possible to study cell-type-specific AEI using scRNA-seq data, the high cost of scRNA-seq has limited its application in studies that involve a large number of individuals. As bulk RNA-seq is more cost-effective and bulk RNA-seq data in clinically relevant tissues are widely available, to better utilize existing bulk RNA-seq data and help refine bulk-level AEI, we developed BSCET, a novel method that can detect cell-type-specific AEI across individuals. BSCET is not aimed to replace the traditional bulk AEI detection methods, but rather is set to help elucidate in which cell types AEI is present. To gain a comprehensive understanding of genetic polymorphisms on gene expression variation, we suggest analyzing the data using both the traditional bulk tissue AEI detection methods and BSCET.

To detect cell-type-specific AEI at the population level, BSCET aggregates information across individuals of the same transcribed SNP. When only RNA-seq data are available, a major challenge for cross-individual AEI detection is the difficulty of aligning allele-specific read counts, as the corresponding regulatory SNP for a given gene is often not observed. As BSCET is designed for analyzing RNA-seq data in the absence of DNA genotypes, therefore in BSCET, we assumed the unobserved regulatory SNP is in complete linkage disequilibrium (LD) with the transcribed SNP, which allows us to align read counts from different individuals based on alleles of the transcribed SNP. We recognized that when the unobserved regulatory SNP and the transcribed SNP are not in complete LD, we will lose power as the individuals will be aligned incorrectly. Therefore we calculated the percentage of individuals who will be incorrectly aligned under a range of minor allele frequency (MAF) and LD correlation coefficient (*r*^2^) between the *cis*-regulatory SNP and the transcribed SNP, and found only a small proportion of the individuals, i.e., < 5%, will be incorrectly aligned in the presence of incomplete LD under the scenarios we investigated (**S6 Table**). The aligned allelic read counts enabled the detection of AEI across individuals at the population level. Through extensive benchmark evaluations, we showed that BSCET had adequate power to detect moderate AEI at the cell type level. Through further applications to human pancreatic islet RNA-seq datasets, we demonstrated that genes with cell-type-specific AEI uncovered by BSCET are biologically relevant to pancreatic functions and the progression of T2D.

However, through benchmark studies, we noticed that the type I error rate of BSCET is conservative. We have examined whether this conservativeness is due to collinearity as cell type proportions sum to 1 since we included all cell types in the benchmark analysis. However, similar type I error rate was observed when we eliminated some cell types from the analysis. Another possibility for the conservativeness could be due to the normality assumption for the allelic count difference in the regression model. When the count of each allele is assumed to follow a Negative Binomial distribution, it is not straightforward to derive the explicit distributional form of allelic count difference. Therefore, in BSCET, we approximate it with a normal distribution assuming sample size is sufficiently large. We believe that modeling the allelic read count difference with a more fitted distribution may further empower BSCET for detecting cell-type-specific AEI, and we will pursue this direction in the future.

We note that when DNA genotype data are available, one can use tools, such as phASER [40], to infer haplotypes and generate haplotype-level allelic read counts. Then based on alleles of a candidate regulatory SNP, individuals can be correctly aligned with respect to the *cis*-regulatory SNP. The haplotype-level read counts aggregate counts across multiple SNPs, which will be higher than SNP-level allelic read counts, and hence will lead to improved power for BSCET.

BSCET uses estimated cell type proportions of bulk RNA-seq data as input. Ideally, if the true cell type proportions are known, we would like to use them in order to obtain an accurate estimate of cell-type-specific AEI. As number of parameters scaled linearly with number of cell types in BSCET, to improve power with limited sample size, we recommend removing rare cell types, e.g., mean proportion < 2%, when detecting cell-type-specific AEI. In the absence of true cell type proportions, we can estimate them using cell type deconvolution methods such as MuSiC [14], which borrows information from external scRNA-seq data to infer the cell type composition of a bulk RNA-seq sample. The benchmark evaluations and real data applications showed that the results obtained using “estimated” cell type proportions were highly correlated with those obtained using true proportions. We further illustrated that, even though different scRNA-seq reference can lead to varied proportion estimates, such variations did not affect the BSCET results much as the detected AEI genes agreed well, suggesting that BSCET is robust to estimation uncertainty on the input.

As a regression-based method, BSCET is flexible and can include covariates in the model and evaluate how covariates would affect cell-type-specific AEI. We have demonstrated BSCET has adequate power to detect association between cell-type-specific AEI with covariates through benchmark evaluations under a variety of scenarios. Further, we applied the extension model to human pancreatic islet bulk RNA-seq data and detected genes whose cell-type-specific AEI is associated with T2D progression. We note that as more parameters are included in the model, a larger sample size is required. Therefore, we would recommend users to perform such analysis as a supplemental step only after evidence of cell-type-specific AEI is detected in at least one of the two groups or in all individuals in the data.

In summary, we have developed BSCET, a regression-based method that integrates bulk RNA-seq and scRNA-seq data to detect cell-type-specific AEI across individuals in a population. BSCET refines the current bulk-level AEI detection workflow and helps understand gene regulation and its association with phenotypic variations across individuals at cell type resolution. As bulk RNA-seq is widely adopted by many biomedical studies and more samples are being sequenced, instead of generating large samples using scRNA-seq, it is desirable for researchers to fully utilize the existing bulk RNA-seq data and learn a comprehensive picture of allelic imbalance in a more cost-effective way. We believe BSCET, will be a great supplemental tool for utilizing the easily accessible bulk sequencing data to elucidate cell type contributions in human diseases.

## Methods

BSCET takes two datasets as input: 1) a bulk RNA-seq dataset in which bulk level gene expression is measured in a relatively large set of individuals; and 2) a scRNA-seq dataset with cells generated from the same tissue as the bulk RNA-seq data in a small set of individuals. The scRNA-seq dataset can be from the same individuals in the bulk RNA-seq dataset, or from different individuals in an external dataset. The goal of BSCET is to detect cell-type-specific AEI in the bulk RNA-seq samples. BSCET involves two steps, and an overview of the procedure is shown in **Fig 1**.

### Step 1: Estimating cell type proportions by deconvolution

Since cell type proportions are often unknown in bulk RNA-seq samples, in this step, we aim to infer cell type proportions in the bulk RNA-seq samples by incorporating cell-type-specific gene expression information provided by a scRNA-seq reference. This can be achieved by cell type deconvolution algorithms such as MuSiC [14]. Following MuSiC [14], for gene *g* of individual *j*, the total bulk-level read count, *X*_*jg*_, can be written as a weighted sum of *K* cell-type-specific gene expressions,

$$X_{jg}=m_{j}\sum_{k=1}^{K}p_{j}^{k}S_{j}^{k}\theta_{jg}^{k},$$

where, for individual *j*, *m*_*j*_ is the total number of cells in the tissue for bulk RNA-seq, $p_{j}^{k}$ is the proportion of cells from cell type *k*, $S_{j}^{k}$ is the average cell size of cell type *k*, and $\theta_{jg}^{k}$ is the relative abundance of gene *g* for cell type *k*. Since subject-level $S_{j}^{k}$ and $\theta_{jg}^{k}$ are often not available, by borrowing information from external scRNA-seq reference, they can be approximated by the sample mean, *S*^*k*^ and $\theta_{g}^{k}$. For individual *j*, as *m*_*j*_ is a constant across all genes, the cell type proportions, $p_{j}^{k}$ (*k* = 1,…*K*), can be inferred through a weighted non-negative least squares regression by regressing the bulk-level expression across all genes, *X*_*jg*_ (*g* = 1,…,*G*), on the cell-type-specific gene expression, ${S^{k}\theta }_{g}^{k}$ (*k* = 1,…*K*). Although we used the estimated cell type proportions obtained from MuSiC [14] for data analyzed in this paper, BSCET is not limited to MuSiC [14] and one can infer cell type compositions using other methods, such as CIBERSORT [41], in this step.

### Step 2: Detecting cell-type-specific AEI

To detect cell-type-specific AEI across individuals in a population, we consider one transcribed SNP at a time. For ease of notation, we omit index for genes. Let *T* and *t* be the two alleles at a transcribed SNP. For individual *j*, let $X_{j}^{T}$ and $X_{j}^{t}$ be the read counts for the *T* and *t* alleles, respectively. Following MuSiC [14], the bulk-level read count of each transcribed allele can be written as a weighted sum of the *K* cell type-specific allelic expressions,

$$X_{j}^{T}=\alpha^{T}+m_{j}\sum_{k=1}^{K}p_{j}^{k}S_{j}^{k}\theta^{kT}+\varepsilon_{j}^{T},$$ (1)

$$X_{j}^{t}=\alpha^{t}+m_{j}\sum_{k=1}^{K}p_{j}^{k}S_{j}^{k}\theta^{kt}+\varepsilon_{j}^{t},$$ (2)

where *α*^*T*^ and *α*^*t*^ are intercepts to capture the information not explained by the *K* cell types, $\varepsilon_{j}^{T}$ and $\varepsilon_{j}^{t}$ are independent random errors assumed to follow *N*(0, *σ*^2^), and *θ*^*kT*^ and *θ*^*kt*^ are mean expression of the transcribed alleles, for cell type *k*, across individuals in the population. Taking difference between (1) and (2), we get

$$X_{j}^{T}-X_{j}^{t}=\alpha +m_{j}\sum_{k=1}^{K}p_{j}^{k}S_{j}^{k}\theta^{k}+\varepsilon_{j},$$ (3)

where *α* = *α*^*T*^−*α*^*t*^ represents allelic expression difference not explained by the selected *K* cell types, and *θ*^*k*^ = *θ*^*kT*^−*θ*^*kt*^ represents allelic expression difference between alleles *T* and *t* in cell type *k*.

We expect the two transcribed alleles to be equally expressed, i.e., *θ*^*k*^ = 0, in the absence of cell-type-specific AEI, and *θ*^*k*^≠0 otherwise. Therefore, to detect cell-type-specific AEI in the population, for cell type *k*, we test the following hypothesis,

$$H_{0}:\;\theta^{k}=0\;versus\;H_{1}:\;\theta^{k}\neq 0$$

using a *t* statistic.

Although we can use estimated cell type proportions obtained from MuSiC [14] in Step 1 to approximate $p_{j}^{k}$, *m*_*j*_ and $S_{j}^{k}$ are not directly observed. To circumvent this, we use the individual-specific library size factor obtained from DESeq2 [42] as an estimate for *m*_*j*_. For $S_{j}^{k}$, we borrow the idea from MuSiC [14] and approximate it by *S*^*k*^, assuming that cell size of all individuals is equal for a given cell type. Since *S*^*k*^ is a constant across all individuals, it is considered as a nuisance parameter and not estimated.

### Step 2 extension: Association between cell-type-specific AEI and covariates

As a regression-based method, we can readily extend it to assess covariate effects on cell-type-specific AEI. For illustration purposes, we only consider one covariate, but it is straightforward to incorporate multiple covariates. Let *V*_*j*_ be the covariate of interest for individual *j*. We modify model (3) by adding an interaction term between the covariate and the estimated cell type proportions:

$$X_{j}^{T}-X_{j}^{t}=\alpha +m_{j}\sum_{k=1}^{K}p_{j}^{k}S_{j}^{k}{(\theta }^{k}+V_{j}\theta_{\Delta }^{k})+\varepsilon_{j},$$ (4)

where *θ*^*k*^ is the population-level AEI of the transcribed SNP for cell type *k* controlling for the covariate; $\theta_{\Delta }^{k}$ is the covariate effect on the cell-type-specific AEI, interpreted as the change in population-level AEI for cell type *k* as the covariate increases by 1 unit; *ε*_*j*_ is the random error term and assumed to follow *N*(0, *σ*^2^). For example, in a case-control study where the covariate indicates disease status (e.g., coded as 1 for cases), *θ*^*k*^ is the cell-type-specific AEI in controls, and $\theta_{\Delta }^{k}$ is the difference in the cell-type-specific AEI between cases and controls, a.k.a. cell-type-specific differential AEI.

In practice we will likely test for the covariate effect on cell-type-specific AEI only if a cell-type-specific AEI has been detected based on model (3). Therefore, in model (4), we are interested in testing whether the cell-type-specific AEI changes with the covariate, i.e., for each cell type *k*, we test the following hypothesis,

$$H_{0}:\;\theta_{\Delta }^{k}=0\;versus\;H_{1}:\;\theta_{\Delta }^{k}\neq 0$$

using a *t* statistic.

### Benchmark bulk RNA-seq data generation

To evaluate the performance of BSCET, we constructed benchmark bulk RNA-seq data in which the cell type proportions and AEI levels for each cell type are known. The benchmark data include 60 heterozygous individuals generated using a publicly available scRNA-seq dataset on human pancreatic islets from three healthy individuals [12]. For each individual in the benchmark bulk RNA-seq data, we generated 5,000 single cells from six selected cell types, including alpha, beta, delta, ductal, gamma and stellate. The cell type proportions were assumed to follow a Dirichlet distribution, with mean proportions inferred from the original scRNA-seq data [12] (**Fig 2A**).

Since BSCET analyzes one transcribed SNP at a time, for the benchmark bulk RNA-seq data, we assumed each gene only has one transcribed SNP. For each cell, we simulated read counts for 11,300 SNPs, corresponding to the number of genes expressed in the original scRNA-seq data. To better reflect patterns seen in real data, for each cell type, we generated read counts only for a fraction of cells, with the percentage of expressed cells inferred from the original scRNA-seq data. **Fig 2B** shows the overall workflow for benchmark dataset generation. Briefly, for each gene, we obtained the mean cell-type-specific expression across cells from the original scRNA-seq data. This mean value set the Poisson distribution rate parameter and subject-specific mean expression was sampled from this Poisson distribution. To generate cell-specific total read count for each individual, we sampled from another layer of Poisson distribution where the rate parameter was determined from the previous step. The total read count of each transcribed SNP was then split into two allele-specific read counts through a binomial distribution with the probability set by the predefined cell-type-specific AEI for the gene. For cell types without AEI, we set their AEI level to 0.5 for all genes. For cell types with AEI, we assumed their AEI levels for each gene followed a truncated gamma distribution with the majority of genes having small to moderate AEI. By summing up allelic read counts across all cells, we obtained the benchmark bulk-level gene expression data.

We also generated benchmark data that include samples from two conditions (e.g. healthy versus diseased). This allows us to evaluate the performance of BSCET in detecting covariate effect on cell-type-specific AEI. Using similar data generation scheme as described above, we generated artificial single-cell and bulk RNA-seq data for 200 individuals, with the ratio of healthy and diseased being 1:1 or 7:3. For each gene, we assumed only the major cell type, i.e., the cell type with the highest mean expression among all cell types, had AEI, and 50% of the genes had differential AEI between healthy and diseased individuals. For genes with cell-type-specific differential AEI, the AEI level for diseased individuals was assumed to be higher than that of healthy individuals by Δ_*AEI*_, which took two possible values, 0.1 and 0.2.

## Supporting information

S1 Fig Mean expression of all tested SNPs at cell type level. Using the benchmark single cell data assuming one cell type with AEI, we draw boxplots of **(A)** cell-type-specific mean expression (at a log scale), and **(B)** molecular proportion, calculated as the cell-type-specific mean expression multiplied by cell type proportion, for each cell type across SNPs. For each cell type, only SNPs with cell-type-specific AEI for the cell type were plotted. We further colored SNPs by whether or not they were detected as having cell-type-specific AEI by BSCET.
(TIF)

S2 Fig Benchmark comparison of BSCET and bulk GLM method assuming one cell type with AEI. From the benchmark data assuming one cell type with AEI, we selected SNPs with small to moderate bulk-level AEI, i.e., < 0.6, and plotted their bulk-level mean expression against the AEI, where the bulk-level AEI was estimated as the reference allele proportion, i.e., the proportion of reference allele read count relative to the total count of both alleles of each SNP. We colored each SNP according to whether it was detected as cell-type-specific AEI by BSCET only (green), AEI by the bulk GLM method only (blue), or by both methods (red). On each margin, using the same color scale, we used histogram to show the distribution of bulk-level AEI **(top)** and mean expression level **(right)** for the SNPs.
(TIF)

S3 Fig Benchmark evaluation for cell-type-specific AEI detection assuming one cell type with AEI. We evaluated the performance of BSCET when only the major cell type for each SNP has AEI using “estimated” cell type proportions, where the “estimated” proportions were obtained by adding random noise to the true proportions. **(A**) Correlation of true cell type proportions versus the “estimated” cell type proportions at cell type level. **(B)** Scatter plot of cell-type-specific AEI p-values obtained using true cell type proportions versus those obtained using “estimated” proportions. **(C)** Type I error rate and power, separated by the cell type and true AEI level, at significance level *α* = 0.05 (dashed line). The solid line indicates the overall power across all cell types at each level of AEI.
(TIF)

S4 Fig Benchmark comparison of BSCET and bulk GLM method assuming two cell types with AEI. The benchmark data were generated assuming the major cell type and a non-major cell type had AEI. Here we focused on the 30% SNPs with opposite AEI directions for the major and non-major cell types, i.e., their AEIs sum to 1, making their AEIs in opposite directions. We selected SNPs with small bulk-level AEI, i.e., within 0.45–0.55, and plotted their bulk-level mean expression against the AEI, where the bulk-level AEI was estimated as the reference allele proportion, i.e., the proportion of reference allele read count relative to the total count of both alleles of each SNP. We colored each SNP according to whether it was detected as cell-type-specific AEI by BSCET only (green), AEI by the bulk GLM method only (blue), or by both methods (red). On each margin, using the same color scale, we used histogram to show the distribution of bulk-level AEI **(top)** and mean expression level **(right)** for the SNPs.
(TIF)

S5 Fig Benchmark evaluation for cell-type-specific AEI detection assuming two cell types with AEI. The benchmark data were generated assuming the major cell type and a non-major cell type had AEI. We evaluated the performance of BSCET using “estimated” cell type proportions, where the “estimated” proportions were obtained by adding random noise to the true proportions. BSCET was evaluated separately for **(A)** SNPs with AEI level for two cell types in the same direction, i.e., both > 0.5 (70%) and **(B)** SNPs with AEI for two cell types in the opposite directions, i.e., sum to 1 (30%). Within each scenario, we compared the SNP-level p-values obtained using true cell type proportions versus those obtained using “estimated” cell type proportions **(left)**, and evaluated the type I error rate and power, separated by the cell type and true AEI level, at significance level *α* = 0.05 (dashed line) for the major **(middle)** and non-major cell type **(right)**. The solid line indicates the overall power across all cell types at each level of AEI.
(TIF)

S6 Fig Benchmark evaluation for cell-type-specific differential AEI (DAEI) detection. We evaluated the performance of BSCET as a function of sample size for healthy (i.e., non-T2D) and diseased (i.e., T2D) samples, and true cell-type-specific AEI difference between healthy and diseased samples (0.1 **(A)** and 0.2 **(B)**) using “estimated” cell type proportions, where the “estimated” proportions were obtained by adding random noise to the true proportions. Within each scenario, we compared the SNP-level p-values obtained using true cell type proportions versus those obtained using the “estimated” proportions through scatter plots **(top)**. And evaluated the type I error rate (non-DAEI) and power (DAEI)), separated by the cell type and level of AEI in the healthy samples, at significance level *α* = 0.05 (dashed line) (**bottom**). The solid line indicates the overall power across all cell types for each level of AEI in the healthy samples.
(TIF)

S7 Fig Selected genes with cell-type-specific AEI of the Fadista pancreatic islets RNA-seq data. We selected 6 genes, *CPA2* **(A)**, *PRSS3* **(B)**, *PPM1K* **(C)**, *SYT11* **(D)**, *ERO1B* **(E)** and *AHR* **(F)** to show their SNP-level p-values of cell-type-specific AEI detection using the Fadista bulk RNA-seq samples [15]. The cell type proportions were obtained using MuSiC [14] based on Segerstolpe single-cell reference [16]. Within each cell type, different shapes represent different SNPs, with red color indicating significant AEI after FDR multiple testing adjustment.
(TIF)

S8 Fig Deconvolution and cell-type-specific AEI detection of the Fadista pancreatic islets RNA-seq data. **(A)** Cell type proportion estimates of the Fadista bulk RNA-seq data [15] using MuSiC [14] when the Baron scRNA-seq data [12] were used as the single-cell reference. For each cell type, the solid line indicates the median cell type proportions across samples. **(B** and **C)** Scatter plots comparing the SNP-level p-values obtained using cell type proportion estimates based on Segerstolpe single-cell reference [16] versus those obtained based on Baron scRNA-seq data [12] across all cell types **(B)** and by each cell type **(C).**
(TIF)

S9 Fig Deconvolution of the Bunt pancreatic islets RNA-seq data. Cell type proportion estimates of the Bunt bulk RNA-seq data [27] using MuSiC [14] when the Segerstolpe scRNA-seq data [16] were used as the single-cell reference. For each cell type, the solid line indicates the median cell type proportions across samples.
(TIF)

S10 Fig Cell type compositions and selected genes with cell-type-specific AEI associated with the progression of T2D in the Fadista pancreatic islets RNA-seq data. **(A)** Scatter plots of HbA1c vs. estimated cell type proportions of the Fadista bulk RNA-seq data [15] for each cell type with a fitted regression line, where the proportions were estimated by MuSiC [14] using the Baron scRNA-seq data [12] as reference. Within each cell type, we further calculated the Spearman’s correlation coefficient and the corresponding p-value between HbA1c and estimated cell type proportions, showed as the subtitle of each plot. **(B** and **C)** We selected 2 genes, *CCDC32* **(B)** and *LARS* **(C)**, to show their SNP-level p-values of the association between HbA1c and cell-type-specific AEI in Fadista samples [15], where we used cell type proportion estimates obtained using two different scRNA-seq datasets: Segerstolpe *et al*. [16] and Baron *et al*. [12]. Within each cell type, different shapes represent different SNPs. Red color indicates a positive correlation (+) between HbA1c and cell-type-specific AEI, with color brightness representing significance level. Similarly, blue color indicates a negative correlation (-) between HbA1c and cell-type-specific AEI, with color brightness representing significance level.
(TIF)

S1 Table SNPs with cell-type-specific AEI in the Fadista data when the Segerstolpe single-cell data were used as reference. We applied BSCET to the Fadista pancreatic islets bulk RNA-seq data [15] using the MuSiC [14] proportion estimates obtained from the Segerstolpe single-cell reference [16]. We detected 283 SNPs across 129 genes with significant cell-type-specific AEI (FDR adjusted p-value < 0.05).
(XLSX)

S2 Table SNPs with cell-type-specific AEI in the Fadista data when the Baron single-cell data were used as reference. We applied BSCET to the Fadista pancreatic islets bulk RNA-seq data [15] using the MuSiC [14] proportion estimates obtained from the Baron single-cell reference [12]. We detected 300 SNPs across 148 genes with significant cell-type-specific AEI (FDR adjusted p-value < 0.05).
(XLSX)

S3 Table SNPs with cell-type-specific AEI in the Bunt data when the Segerstolpe single-cell data were used as reference. We applied BSCET to the Bunt pancreatic islets bulk RNA-seq data [27] using the MuSiC [14] proportion estimates obtained from the Segerstolpe single-cell reference [16]. We detected 678 SNPs across 405 genes with significant cell-type-specific AEI (FDR adjusted P-value < 0.05).
(XLSX)

S4 Table SNPs with cell-type-specific AEI associated with HbA1c in the Fadista data when the Segerstolpe data were used as reference. We applied BSCET to the Fadista pancreatic islets bulk RNA-seq data [15] using the MuSiC [14] proportion estimates obtained from the Segerstolpe single-cell reference [16]. We detected 8 SNPs in 8 genes with cell-type-specific AEI significantly associated with HbA1c (FDR adjusted P-value < 0.05), with the direction of the association indicated in column ‘Correlation’.
(XLSX)

S5 Table SNPs with cell-type-specific AEI associated with HbA1c in the Fadista data when the Baron data were used as reference. We applied BSCET to the Fadista pancreatic islets bulk RNA-seq data [15] using the MuSiC [14] proportion estimates obtained from the Baron single-cell reference [12]. We detected 5 SNPs in 4 genes with cell-type-specific AEI significantly associated with HbA1c (FDR adjusted P-value < 0.05), with the direction of the association indicated in column ‘Correlation’.
(XLSX)

S6 Table Proportion of misaligned samples in the presence of incomplete LD. We calculated the proportion of samples that are aligned incorrectly in the presence of incomplete LD as a function of the LD correlation coefficient (*r*^2^) between the *cis*-regulatory SNP and the transcribed SNP and the minor allele frequency (MAF) of each SNP.
(XLSX)
