# Supplementary figures and images for: Detecting cell-type-specific allelic expression imbalance by integrative analysis of bulk and single-cell RNA sequencing data
Source: PLoS Genet. 2021 Mar 4;17(3):e1009080. doi: 10.1371/journal.pgen.1009080 (PMC7963069; doi:10.1371/journal.pgen.1009080)

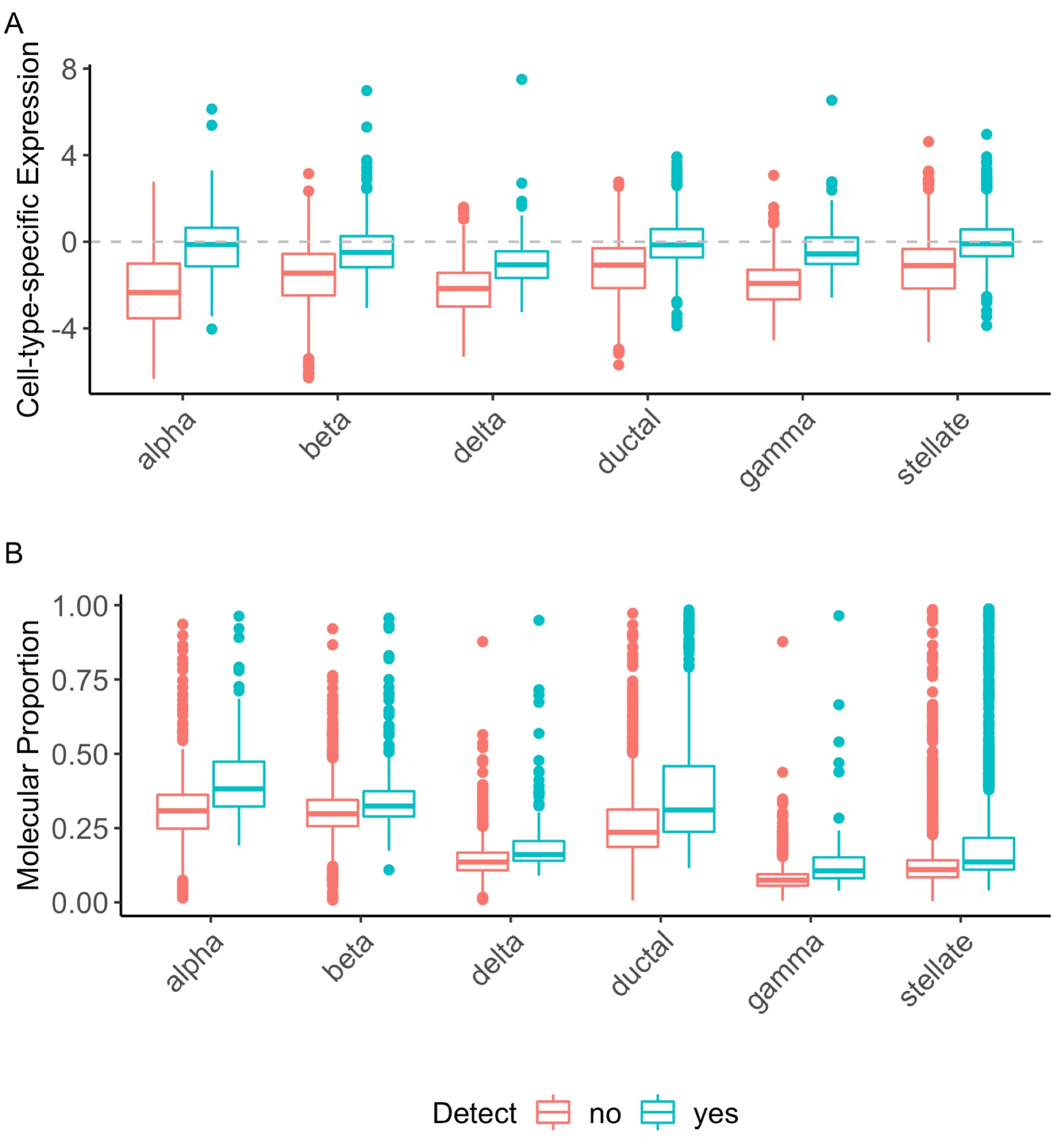

Supplement: S1 Fig — Using the benchmark single cell data assuming one cell type with AEI, we draw boxplots of (A) cell-type-specific mean expression (at a log scale), and (B) molecular proportion, calculated as the cell-type-specific mean expression multiplied by cell type proportion, for each cell type across SNPs. For each cell type, only SNPs with cell-type-specific AEI for the cell type were plotted. We further colored SNPs by whether or not they were detected as having cell-type-specific AEI by BSCET. (TIF) [file pgen.1009080.s001.tif]

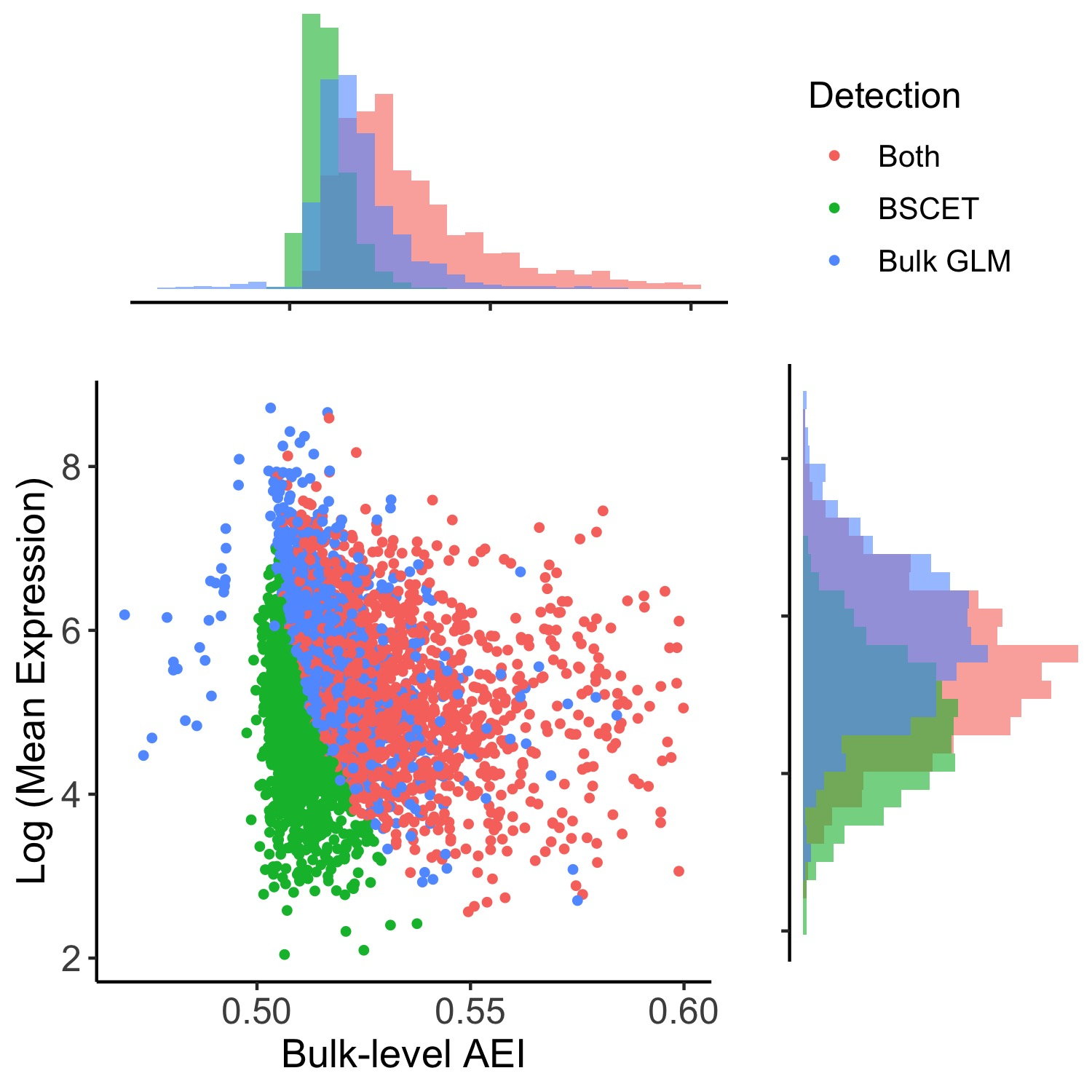

Supplement: S2 Fig — From the benchmark data assuming one cell type with AEI, we selected SNPs with small to moderate bulk-level AEI, i.e., < 0.6, and plotted their bulk-level mean expression against the AEI, where the bulk-level AEI was estimated as the reference allele proportion, i.e., the proportion of reference allele read count relative to the total count of both alleles of each SNP. We colored each SNP according to whether it was detected as cell-type-specific AEI by BSCET only (green), AEI by the bulk GLM method only (blue), or by both methods (red). On each margin, using the same color scale, we used histogram to show the distribution of bulk-level AEI (top) and mean expression level (right) for the SNPs. (TIF) [file pgen.1009080.s002.tif]

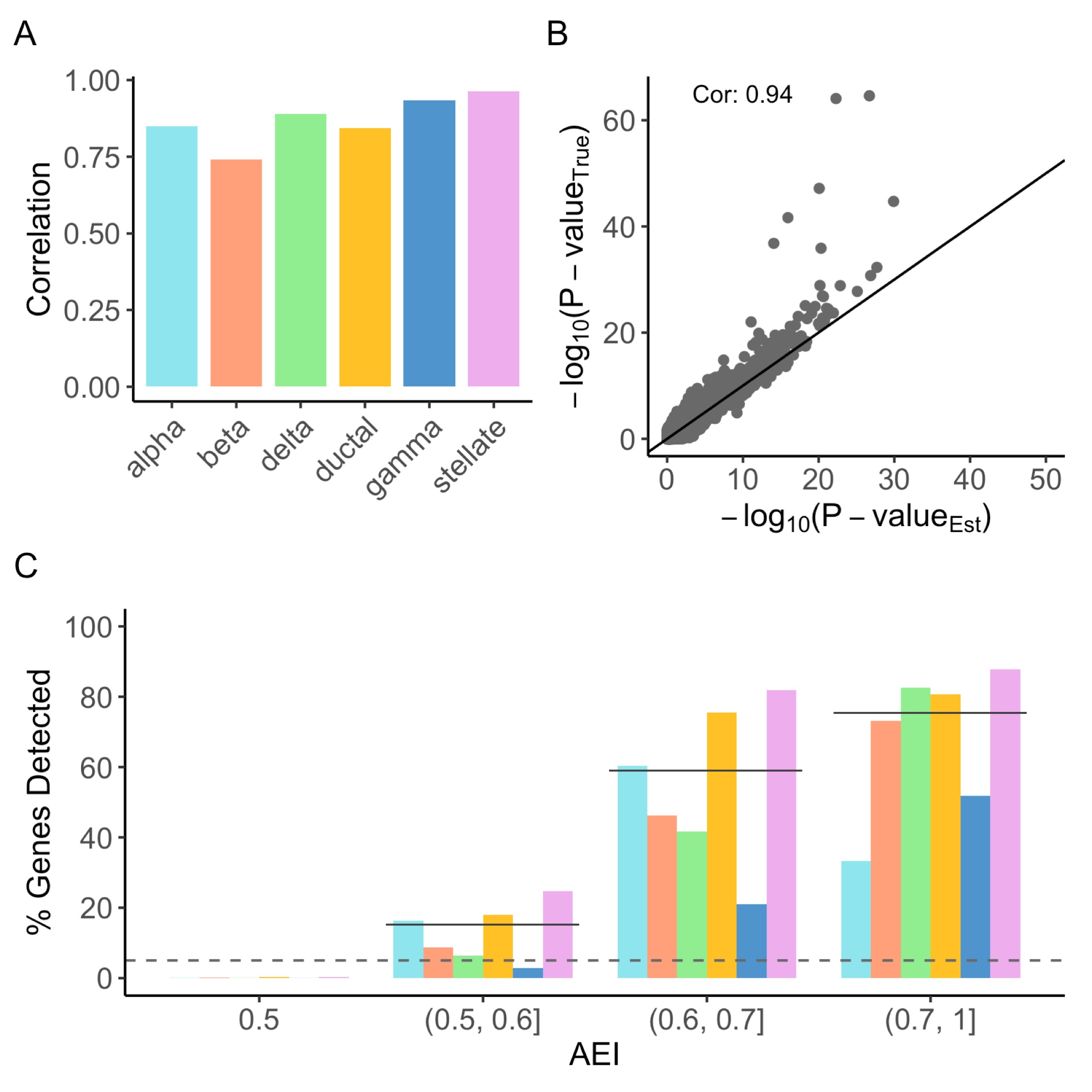

Supplement: S3 Fig — We evaluated the performance of BSCET when only the major cell type for each SNP has AEI using “estimated” cell type proportions, where the “estimated” proportions were obtained by adding random noise to the true proportions. (A) Correlation of true cell type proportions versus the “estimated” cell type proportions at cell type level. (B) Scatter plot of cell-type-specific AEI p-values obtained using true cell type proportions versus those obtained using “estimated” proportions. (C) Type I error rate and power, separated by the cell type and true AEI level, at significance level α = 0.05 (dashed line). The solid line indicates the overall power across all cell types at each level of AEI. (TIF) [file pgen.1009080.s003.tif]

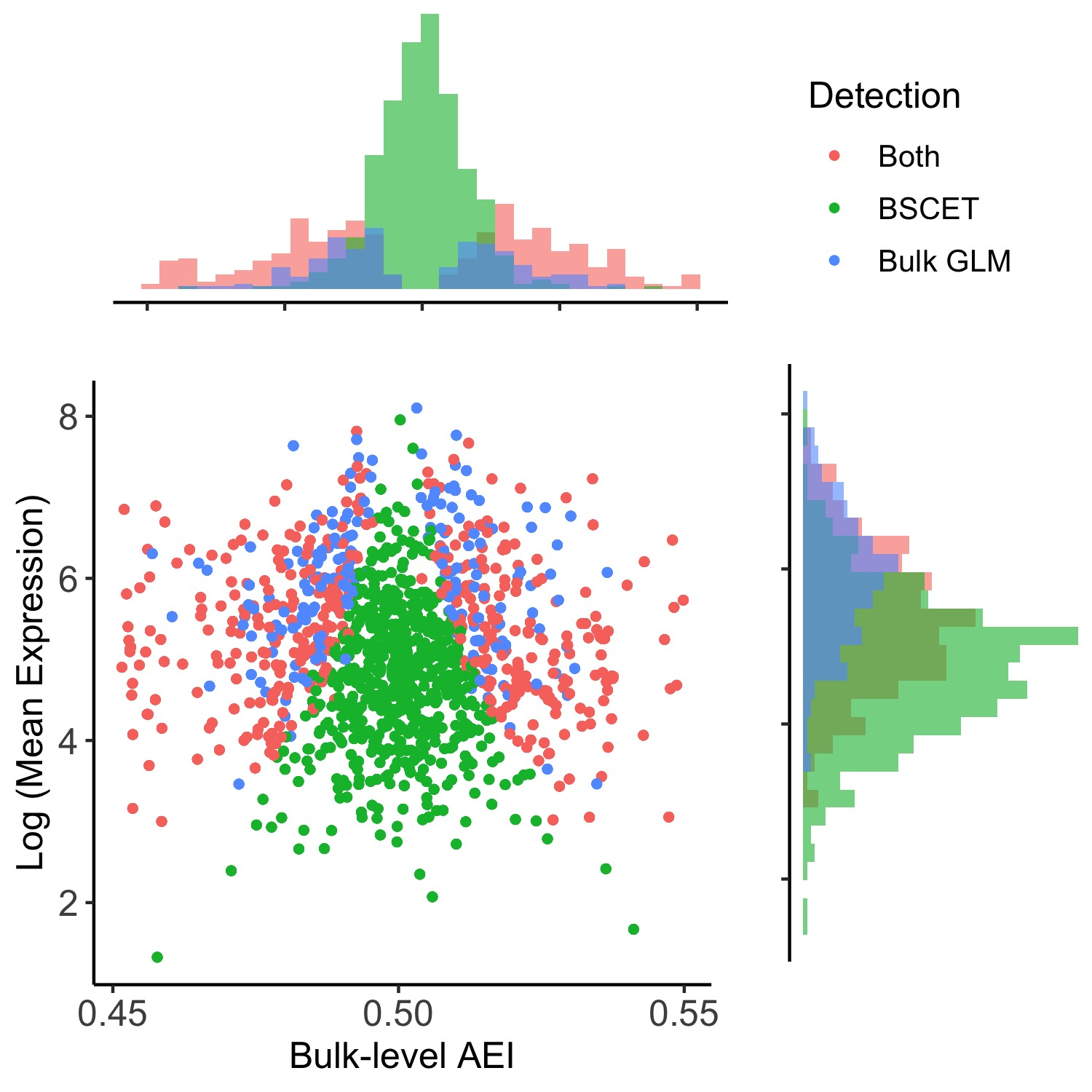

Supplement: S4 Fig — The benchmark data were generated assuming the major cell type and a non-major cell type had AEI. Here we focused on the 30% SNPs with opposite AEI directions for the major and non-major cell types, i.e., their AEIs sum to 1, making their AEIs in opposite directions. We selected SNPs with small bulk-level AEI, i.e., within 0.45–0.55, and plotted their bulk-level mean expression against the AEI, where the bulk-level AEI was estimated as the reference allele proportion, i.e., the proportion of reference allele read count relative to the total count of both alleles of each SNP. We colored each SNP according to whether it was detected as cell-type-specific AEI by BSCET only (green), AEI by the bulk GLM method only (blue), or by both methods (red). On each margin, using the same color scale, we used histogram to show the distribution of bulk-level AEI (top) and mean expression level (right) for the SNPs. (TIF) [file pgen.1009080.s004.tif]

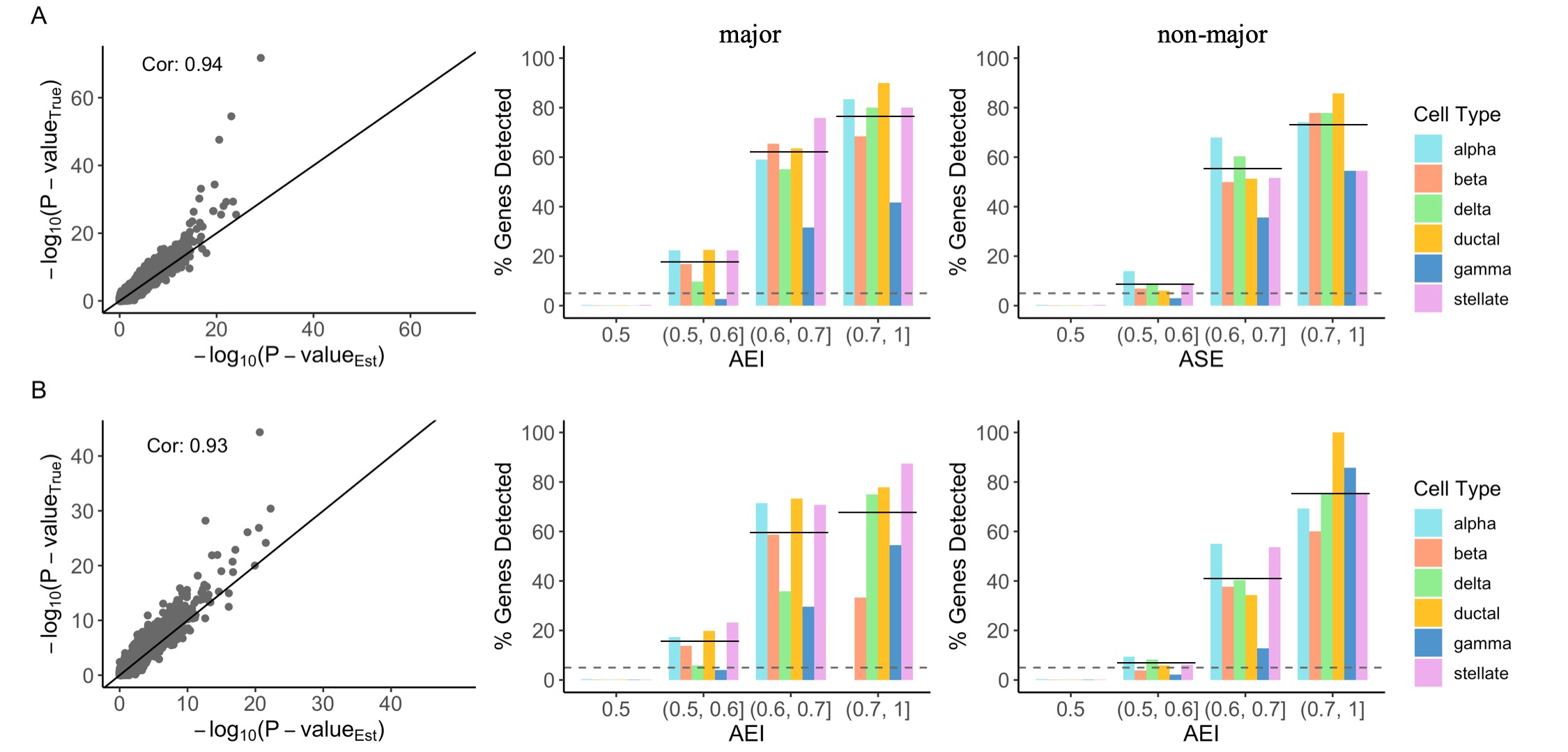

Supplement: S5 Fig — The benchmark data were generated assuming the major cell type and a non-major cell type had AEI. We evaluated the performance of BSCET using “estimated” cell type proportions, where the “estimated” proportions were obtained by adding random noise to the true proportions. BSCET was evaluated separately for (A) SNPs with AEI level for two cell types in the same direction, i.e., both > 0.5 (70%) and (B) SNPs with AEI for two cell types in the opposite directions, i.e., sum to 1 (30%). Within each scenario, we compared the SNP-level p-values obtained using true cell type proportions versus those obtained using “estimated” cell type proportions (left), and evaluated the type I error rate and power, separated by the cell type and true AEI level, at significance level α = 0.05 (dashed line) for the major (middle) and non-major cell type (right). The solid line indicates the overall power across all cell types at each level of AEI. (TIF) [file pgen.1009080.s005.tif]

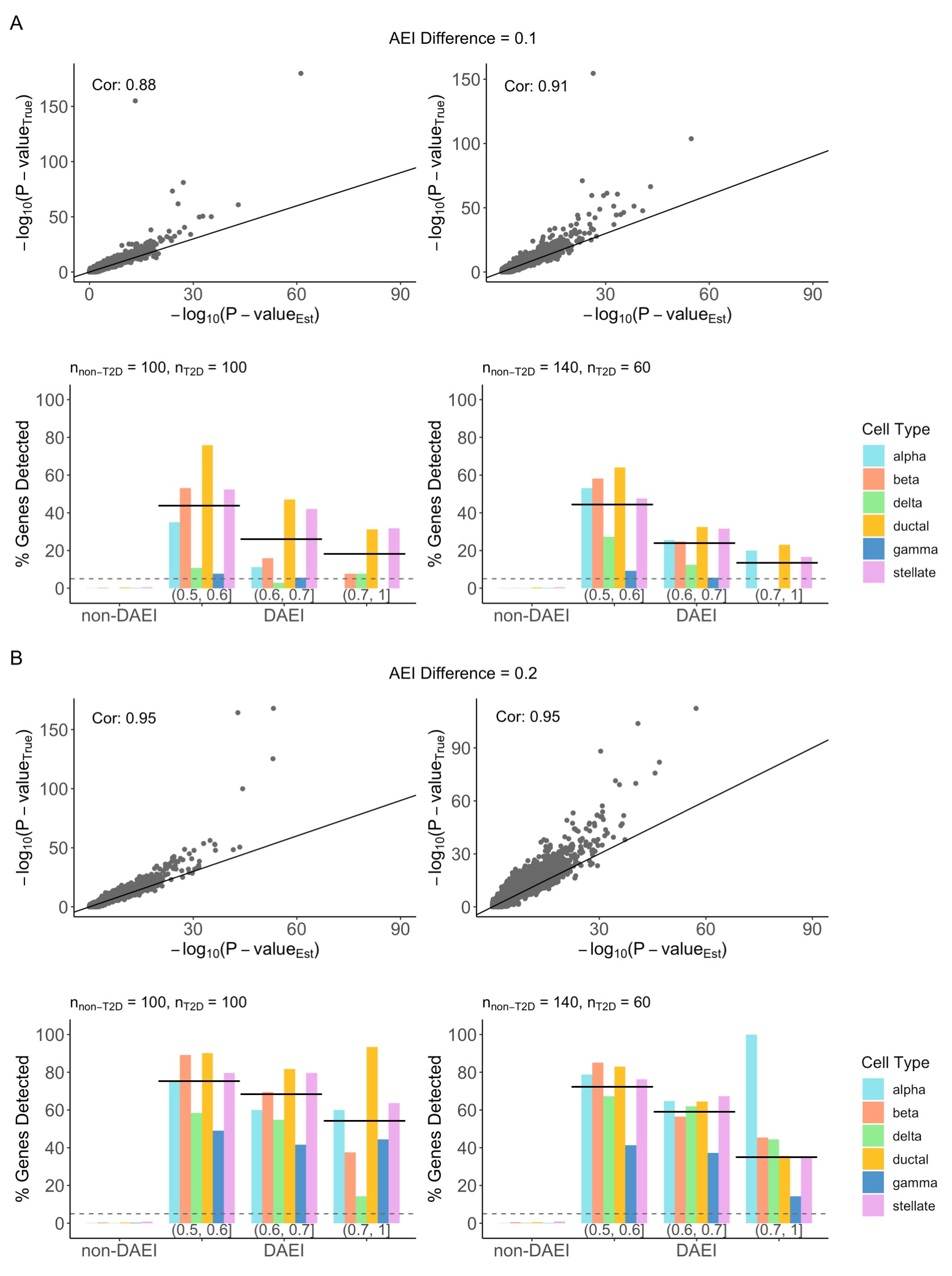

Supplement: S6 Fig — We evaluated the performance of BSCET as a function of sample size for healthy (i.e., non-T2D) and diseased (i.e., T2D) samples, and true cell-type-specific AEI difference between healthy and diseased samples (0.1 (A) and 0.2 (B)) using “estimated” cell type proportions, where the “estimated” proportions were obtained by adding random noise to the true proportions. Within each scenario, we compared the SNP-level p-values obtained using true cell type proportions versus those obtained using the “estimated” proportions through scatter plots (top). And evaluated the type I error rate (non-DAEI) and power (DAEI)), separated by the cell type and level of AEI in the healthy samples, at significance level α = 0.05 (dashed line) (bottom). The solid line indicates the overall power across all cell types for each level of AEI in the healthy samples. (TIF) [file pgen.1009080.s006.tif]

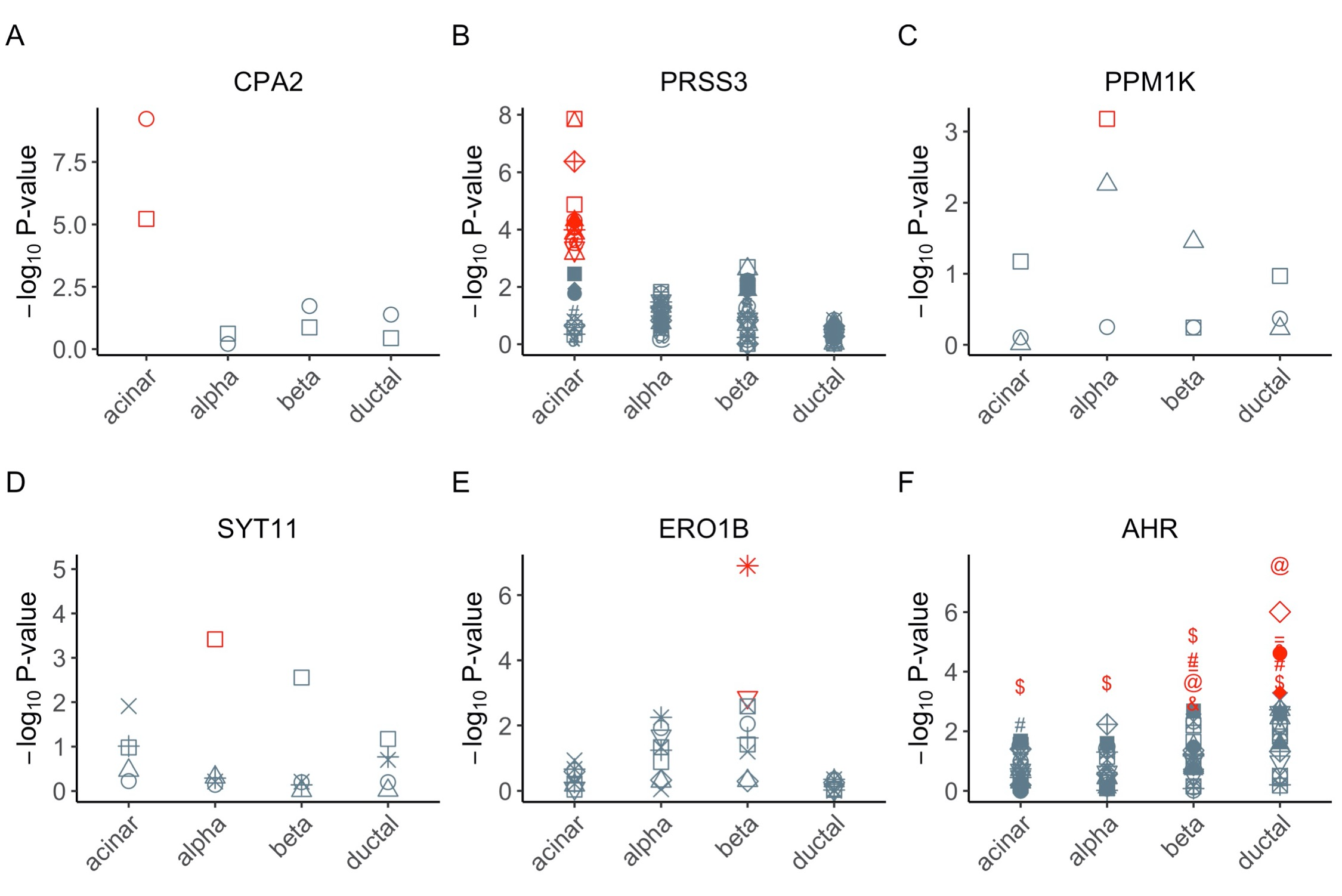

Supplement: S7 Fig — We selected 6 genes, CPA2 (A), PRSS3 (B), PPM1K (C), SYT11 (D), ERO1B (E) and AHR (F) to show their SNP-level p-values of cell-type-specific AEI detection using the Fadista bulk RNA-seq samples [15]. The cell type proportions were obtained using MuSiC [14] based on Segerstolpe single-cell reference [16]. Within each cell type, different shapes represent different SNPs, with red color indicating significant AEI after FDR multiple testing adjustment. (TIF) [file pgen.1009080.s007.tif]

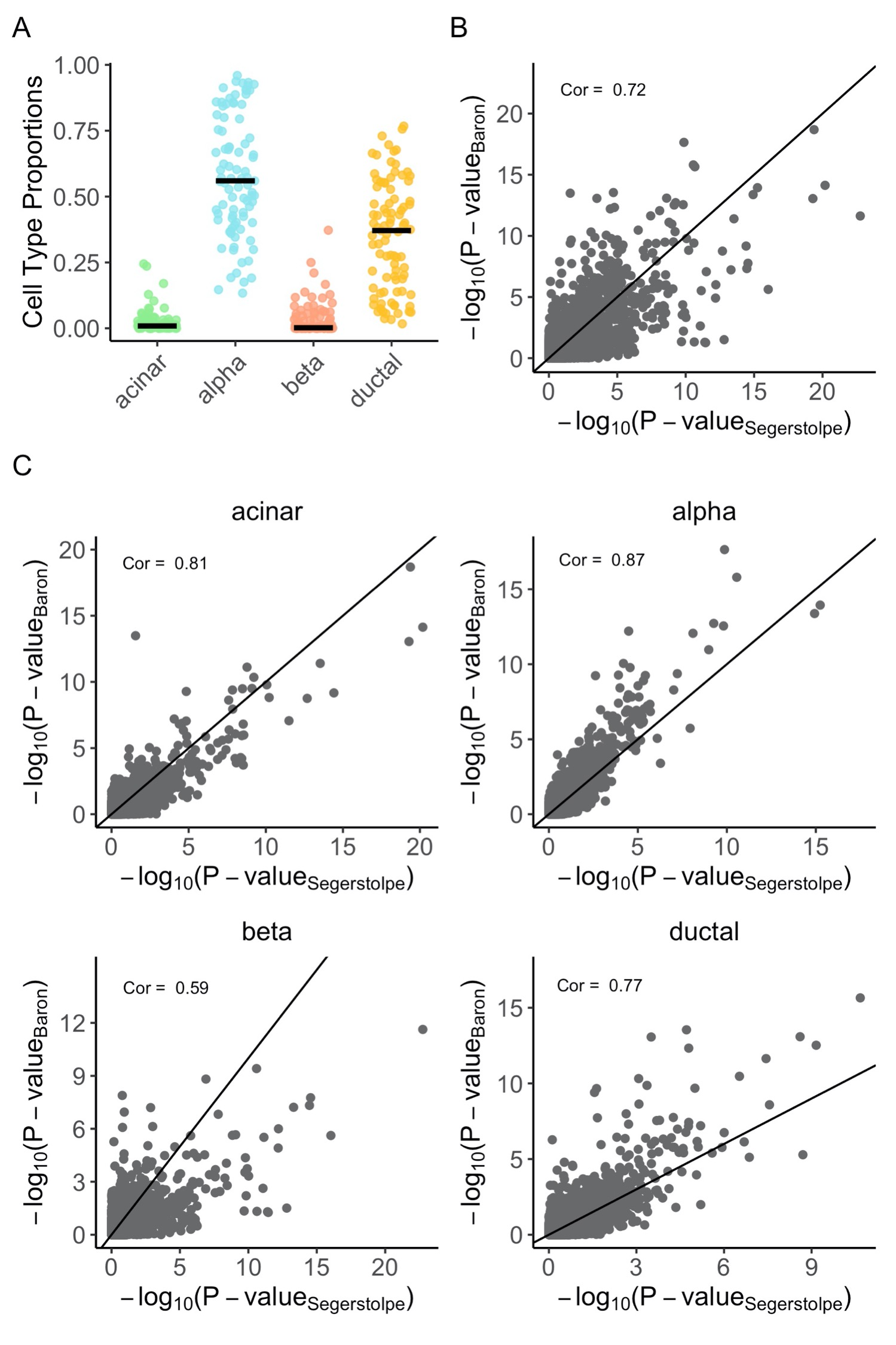

Supplement: S8 Fig — (A) Cell type proportion estimates of the Fadista bulk RNA-seq data [15] using MuSiC [14] when the Baron scRNA-seq data [12] were used as the single-cell reference. For each cell type, the solid line indicates the median cell type proportions across samples. (B and C) Scatter plots comparing the SNP-level p-values obtained using cell type proportion estimates based on Segerstolpe single-cell reference [16] versus those obtained based on Baron scRNA-seq data [12] across all cell types (B) and by each cell type (C). (TIF) [file pgen.1009080.s008.tif]

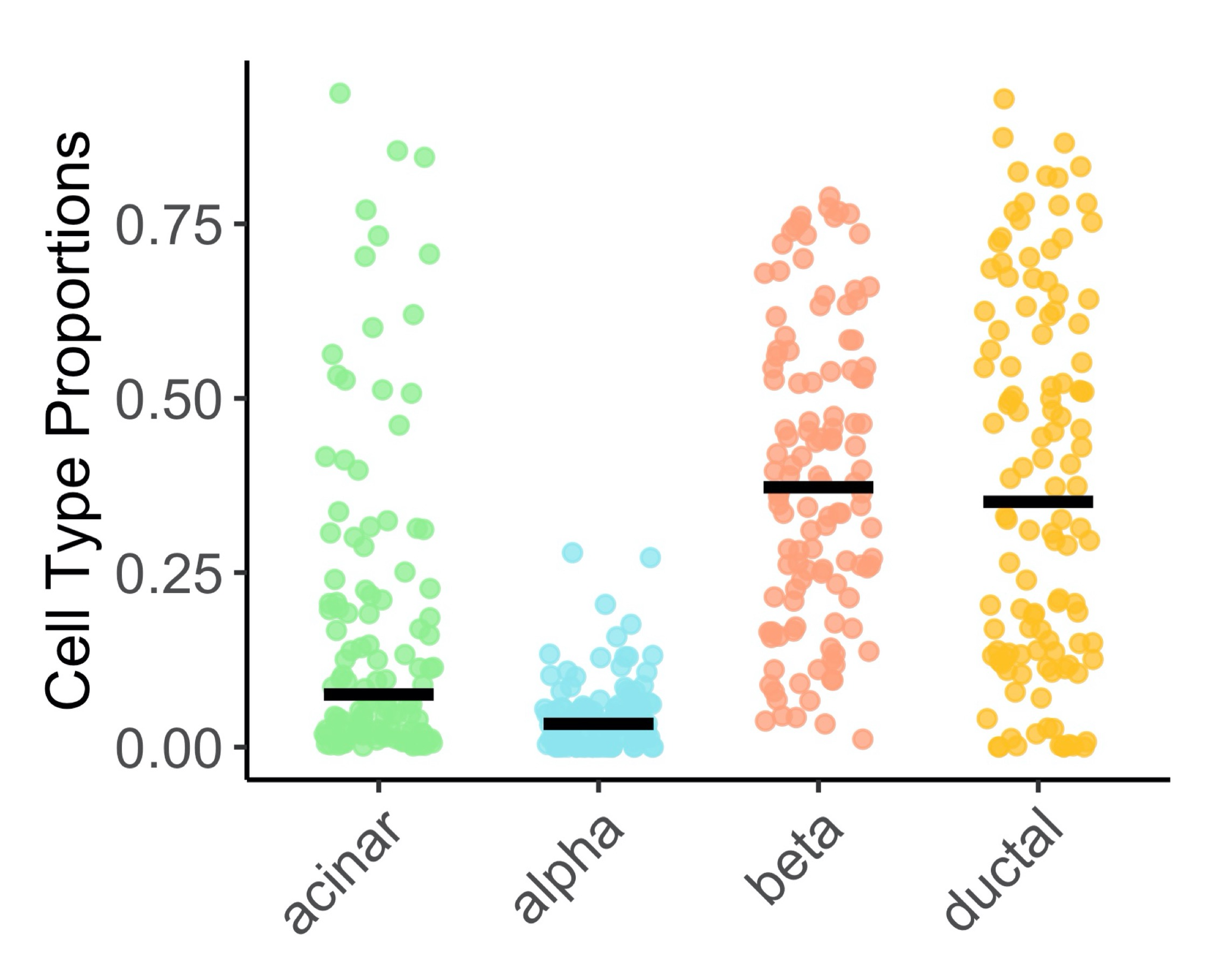

Supplement: S9 Fig — Cell type proportion estimates of the Bunt bulk RNA-seq data [27] using MuSiC [14] when the Segerstolpe scRNA-seq data [16] were used as the single-cell reference. For each cell type, the solid line indicates the median cell type proportions across samples. (TIF) [file pgen.1009080.s009.tif]

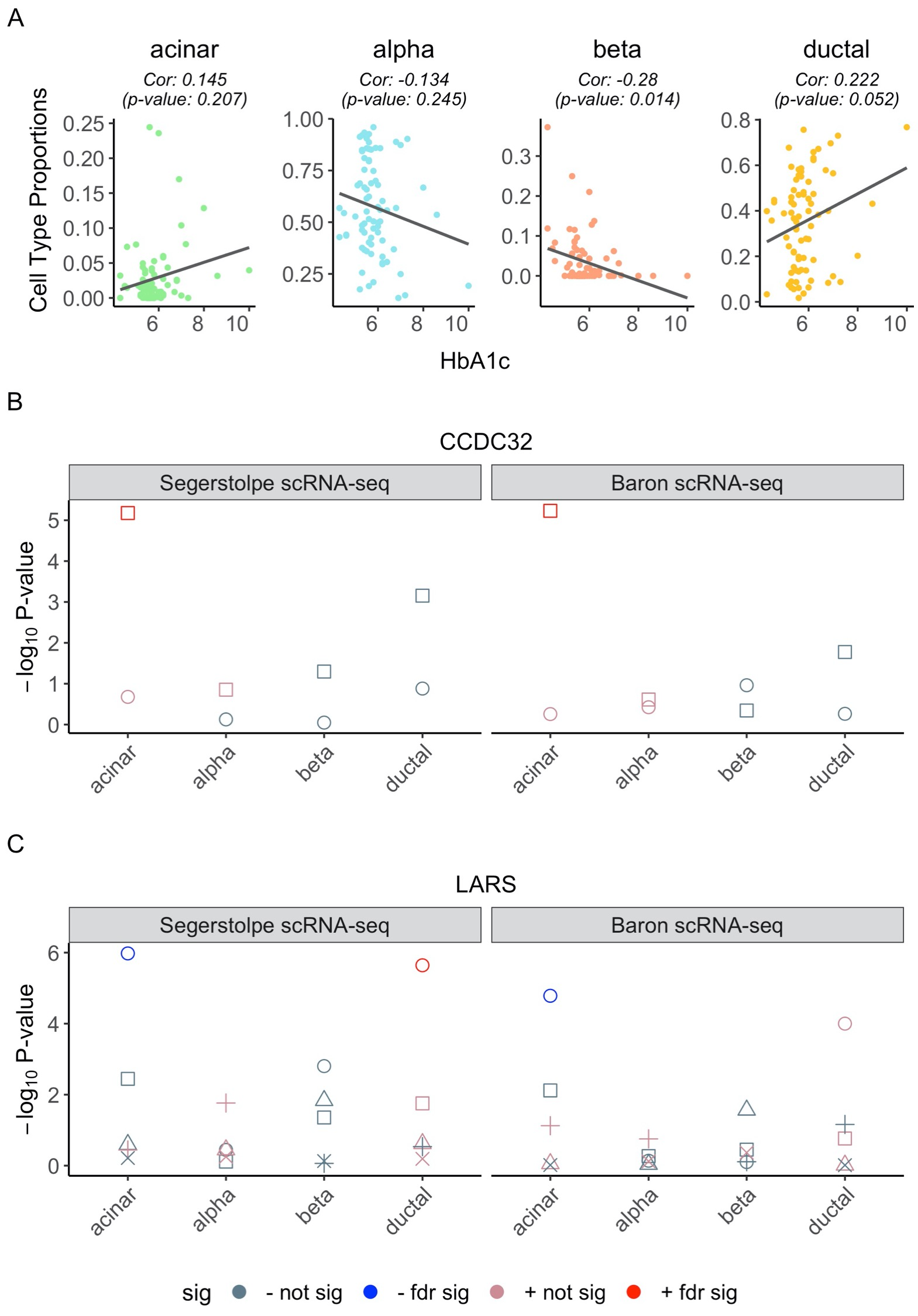

Supplement: S10 Fig — (A) Scatter plots of HbA1c vs. estimated cell type proportions of the Fadista bulk RNA-seq data [15] for each cell type with a fitted regression line, where the proportions were estimated by MuSiC [14] using the Baron scRNA-seq data [12] as reference. Within each cell type, we further calculated the Spearman’s correlation coefficient and the corresponding p-value between HbA1c and estimated cell type proportions, showed as the subtitle of each plot. (B and C) We selected 2 genes, CCDC32 (B) and LARS (C), to show their SNP-level p-values of the association between HbA1c and cell-type-specific AEI in Fadista samples [15], where we used cell type proportion estimates obtained using two different scRNA-seq datasets: Segerstolpe et al. [16] and Baron et al. [12]. Within each cell type, different shapes represent different SNPs. Red color indicates a positive correlation (+) between HbA1c and cell-type-specific AEI, with color brightness representing significance level. Similarly, blue color indicates a negative correlation (-) between HbA1c and cell-type-specific AEI, with color brightness representing significance level. (TIF) [file pgen.1009080.s010.tif]
